# Atomic Clusters: Structure, Reactivity, Bonding, and Dynamics

**DOI:** 10.3389/fchem.2021.730548

**Published:** 2021-08-16

**Authors:** Ranita Pal, Arpita Poddar, Pratim Kumar Chattaraj

**Affiliations:** ^1^ Advanced Technology Development Centre, Indian Institute of Technology Kharagpur, Kharagpur, India; ^2^ Department of Chemistry, Indian Institute of Technology Kharagpur, Kharagpur, India; ^3^ Department of Chemistry, Indian Institute of Technology Bombay, Mumbai, India

**Keywords:** aromaticity, Electrides, Particle swarm optimization, Firefly algorithm, Confinement, Hydrogen storage, Fluxionality

## Abstract

Atomic clusters lie somewhere in between isolated atoms and extended solids with distinctly different reactivity patterns. They are known to be useful as catalysts facilitating several reactions of industrial importance. Various machine learning based techniques have been adopted in generating their global minimum energy structures. Bond-stretch isomerism, aromatic stabilization, Rener-Teller effect, improved superhalogen/superalkali properties, and electride characteristics are some of the hallmarks of these clusters. Different all-metal and nonmetal clusters exhibit a variety of aromatic characteristics. Some of these clusters are dynamically stable as exemplified through their fluxional behavior. Several of these cluster cavitands are found to be agents for effective confinement. The confined media cause drastic changes in bonding, reactivity, and other properties, for example, bonding between two noble gas atoms, and remarkable acceleration in the rate of a chemical reaction under confinement. They have potential to be good hydrogen storage materials and also to activate small molecules for various purposes. Many atomic clusters show exceptional opto-electronic, magnetic, and nonlinear optical properties. In this Review article, we intend to highlight all these aspects.

## Introduction

A cluster is defined as a finite aggregation of atoms, starting with as low as two atoms and extending up to an upper bound of some hundred thousand atoms (Herr, 1993; Corrigan and Dehnen, 2017). A theoretical study elucidates that cluster properties strongly depend on the geometry of the isolated cluster and the topology of the cluster sample. F. A. Cotton first used the term cluster for compounds with metal–metal bonds. Nonmetallic atomic clusters came into the limelight later, from both theoretical and experimental studies. The unique size-dependent properties of clusters are distinct from the molecules and bulk solids (Sergeeva et al., 2014). Experimental and theoretical methods on cluster research have seen substantial amount of improvement over the years in discovering a diversity of size-specific phenomena and physicochemical cluster properties (Jena and Castleman, 2010). Various physicochemical properties of metal clusters such as optical, magnetic, thermal, chemical properties differ remarkably from their bulk counterparts (Tsukuda and Hakkinen, 2015). In the last few decades, several studies have been reported on the ligand protected metal clusters, *viz*. phosphine-protected small Au cluster, thiolate (RS)-protected Au nanoparticles (Brust et al., 1994), *etc*. For small metal clusters (<∼100 atoms), the electronic structures are not continuous as in the bulk metals, but rather discretized, which is the primary reason for different physicochemical properties and functionalities in small clusters and bulk metal (Sharma et al., 2017). Studies have shown that clusters made of atoms with appropriate size and composition could potentially mimic the chemistry of elemental atoms in periodic table, and hence are known as superatoms (Li et al., 2008). Various experimental techniques such as laser ablation coupled with mass spectrometry, photoelectron spectroscopy have been employed to get insights into the atomic clusters. Along with experimental studies, theoretical investigations are required to get a better understanding of their geometric arrangement and corresponding properties (Srivastava, 2021).

A large number of theoretical studies have been reported dealing with finding the minimum energy structures of pure elemental clusters by using different optimization algorithms. The potential energy surface (PES) of various atomic clusters is explored to minimize their energy functional with the final objective of locating their global minimum (GM). Different optimization methods such as genetic algorithm (Holland, 1992) (GA), basin hopping (BH) algorithm (Wales and Doye, 1997), particle swarm optimization (PSO) algorithm (Bai, 2010), adaptive particle swarm optimization (APSO) (Zhan and Zhang, 2008), simulated annealing (SA) (Woodley et al., 1999), artificial bee colony optimization (ABC) (Karaboga and Basturk, 2007), honey bee mating optimization (HBMO) (Pham et al., 2005), ant colony optimization (ACO) (Colorni et al., 1991), heuristic algorithm combined with the surface and interior operators (HA-SIO), fast annealing evolutionary algorithm (FAEA), firefly algorithm (FA) (Yang, 2010). *etc*. are increasingly being used to solve the optimization problem in a time- and cost-efficient manner. Various models/empirical potentials (EPs) such as Lennard–Jones (LJ), Born–Mayer, Sutton–Chen, Gupta and Murrell–Mottram potentials can effectively explain the bonding within various clusters. A number of studies performed by Chattaraj *et al*. reveal that PSO is more efficient than commonly used techniques such as GA, SA, and BH for finding the GM of small clusters (Mitikiri et al., 2018; Jana et al., 2019). Further developments over PSO algorithm have been accomplished. Global optimization of boron clusters (B_5_ and B_6_) has been studied using an advanced PSO approach by Mitikiri et al. (2018). Jana et al. (2019) performed a similar study on carbon clusters, C_*n*_ (*n* = 3–6,10) by using a modified PSO algorithm. Mitra et al. (2020) reported the global optimization of Al_4_^2-^ clusters by using firefly algorithm along with DFT.

The concept of trapping atoms and small molecules into the hollow cavity of clusters has shown several applications in biology (Cagle et al., 1996; Thrash et al., 1999; Wilson et al., 1999) and electrical engineering (Cioslowski and Nanayakkara, 1992). Pan et al. (2018) reported the encapsulation of noble gas (Ng) atoms into the B_40_ host moiety, which is shown to have a fluxional character (Moreno et al., 2014). The dynamical study of the aforementioned system showed that the fluxional character persists even after the encapsulation. Smaller cages such as C_20_H_20_ (Cross et al., 1999; Jiménez-Vázquez et al., 2001), C_10_H_16_ (Haaland et al., 2004), BN cages (B_12_N_12_ and B_16_N_16_) (Khatua et al., 2014a), Pb_12_^2-^, and Sn_12_^2-^ (Sekhar et al., 2017) can act as host molecules to endohedrally trapped noble gas atoms. Cucurbit[*n*]urils, abbreviated as CB[*n*]s, *n* being the number of gycoluril units, can also act as host for different guest molecules including metal cations, organic dyes, drugs, halide ions, *etc.* (Lagona et al., 2005; Pan et al., 2013a). CB[7] was also reported to bind different guest compounds such as organic dyes (*e.g*., Stilbenes, naphthalene), viologens, and metal complexes (*e.g*., Oxaliplatin) applicable in cancer treatment (Wagner et al., 2003). In 2017, Pan et al. (2017) have reported the adsorption of 14 molecules, *viz*., CH_4_, C_2_H_2_, C_2_H_4_, C_2_H_6_, F_2_, Cl_2_, NO_2_, NO, CO, CO_2_, SO_2_, H_2_S, N_2_, H_2_ endohedrally within the hydrophobic inner cavity of CB[7]. CB[6] is also known to encapsulate noble gas atoms (Pan et al., 2015). Chakraborty *et al.* (Chakraborty and Chattaraj, 2015) reported the accommodation of noble gas atoms within the BN-doped (3, 3) single-walled carbon nanotubes. The Ng binding ability of BeX (X = SO_4_, CO_3_, O) has been reported by Saha et al. (2015). Pan et al. (2014) have explored the stability of Ng-bound SiH_3_^+^ cluster ions. An emerging host molecule is the basket-shaped octa acid (OA) cavitand that can encapsulate different gas molecules (Chakraborty et al., 2016). Various steroids (Liu and Gibb, 2008), hydrophobic moieties such as ethane, ethylene, acetylene (Stang and Diederich, 2008; Hu et al., 2009; Florea and Nau, 2011; Zhang et al., 2011; Yang et al., 2015; Chong et al., 2016) have been confined inside OA. The encapsulation of gas molecules, especially the hazardous ones, by the cluster cavity has great applications in environmental chemistry. Encapsulation of greenhouse gases (CO_2_) (Zhang and Chen, 2009; Jin et al., 2010; Kim et al., 2010; Jin et al., 2011; Mastalerz et al., 2011; Lü et al., 2014), air pollutants (NO_2_) (Pan et al., 2017), and poisonous gases (CO, NO) using molecular cages has applications in reducing their negative impact on the atmosphere. N_2_ encapsulation (Msayib et al., 2009; Akhtar et al., 2012; Schneider et al., 2012) is yet another important research topic in environmental chemistry. Chakraborty et al. (2016) reported a set of small gaseous molecules (C_2_H_2_, C_2_H_4_, C_2_H_6_, CO, CO_2_, NO_2_, NO, N_2_, H_2_^−^), and rare gas atoms as guest molecules for the OA host system. In fact, OA is a very efficient reaction vessel for accommodating various different guest molecules. Li^+^, Na^+^, K^+^, Be^2+^, Mg^2+^, Ca^2+^, Li_3_O^+^, Na_3_O^+^, K_3_O^+^ and various nucleobases can occupy the basket-shaped octa acid cavitand as reported by Chakraborty and Chattaraj (2018).

In recent days, the conservation of the atmosphere and the desire to save up fuel for the upcoming generations has been a major concern in the scientific community that led to the search for alternatives of fossil fuel. Hydrogen, being renewable, recyclable, environment friendly, and abundantly available in nature, is now a globally acceptable fuel source with the potential to replace fossil fuels in the near future. The challenge, however, is designing compatible storage and transport materials. To that end, hydrogen-storing capacity of metal-organic-frameworks (MOF) (Rosi et al., 2003; Rowsell and Yaghi, 2005), covalent-organic-frameworks (COF) (Kuc et al., 2007; Cabria et al., 2008), clathrate hydrates (Lee et al., 2005; Chattaraj et al., 2011), polymers (McKeown et al., 2006), carbon nanotubes, BN cages, fullerene, grapheme-like materials (Froudakis, 2001; Deng et al., 2004; Heine et al., 2004; Sun et al., 2005; Wu et al., 2008), metal hydrides (Lee et al., 2005) have been explored. Pan et al. (2012a) performed a theoretical study on the H_2_-storing capability of some Li-doped clusters and super-alkalis. Zhu et al. (2010) have shown cucurbiturils acting as a promising candidate for hydrogen storage. Pan et al. (2013a) have discussed the hydrogen storage capability of the CB[7] system. A different class of compound, alkali-doped carbon materials (graphene sheet and single-walled carbon nanotubes), have been designed for reversible hydrogen storage for transportation purposes by Wei-Qiao Deng et al. (2004). On the other hand, very explosive acetylene can be stored within the porous MOF-505 analogue as reported by Yunxia Hu et al. (2009).

The confinement effect on atoms and molecules has intrigued both theoreticians and experimentalists alike. It brings out interesting changes in the energy levels of the confined systems, their bonding, reactivity, and properties (Grochala et al., 2007; Schettino and Bini, 2007; Sabin and Brandas, 2009; Gubbins et al., 2011; Chakraborty and Chattaraj, 2019; Pal and Chattaraj, 2021). Khatua et al. (2014b) have performed a theoretical investigation on the entrapment of (HF)_2_ in C_*n*_ (*n* = 60, 70, 80, 90) cages. Although CO and N_2_ are isoelectric species, the latter is known to be pretty inert owing to its high ionization potential, low electron affinity, and high frontier orbital energy gap (Δ*E*_HOMO-LUMO_). Thus, N_2_ capture in various transition metal complexes has proven to induce bond activation that has various industrial applications (Chatt and Leigh, 1972; Yandulov and Schrock, 2003; Latysheva et al., 2012; Bergman et al., 2013; Hoffman et al., 2013). In this regard, Saha et al. (2017) reported the CO and N_2_ bound metal supported boron clusters (MB_12_^-^, M = Co, Rh, Ir) which form a spinning umbrella-like structure and activate the bound molecules. Boron clusters have found profound applications in material science owing to their ability to act as nanomaterial building blocks. Their property to act as such is due to a bowl-like structure with an outer rim (B_9_) and an inner well (B_3_).

Along with the coordination and inorganic cages, various organic cavitands can also be considered host system for encapsulation. One such class of compounds is the cucurbiturils. Chemical reactions catalyzed by host–guest interactions are comparable to those catalyzed by enzymes (Dong et al., 2012). Hennig et al. (2007) successfully induced protease inhibition using the host–guest interaction with CB[7]. In addition to CB[7], ExBox^+4^ (Barnes et al., 2013) can also act as an organic host molecule that can encapsulate a wide array of guest moieties. Chakraborty et al. (2017) performed a theoretical study on [4+2] cycloaddition reaction confined within CB[7] and ExBox^+4^ host systems.

In recent times, low dimensional materials are being given more and more attention to be used as host moiety. Graphene has provided us with a plethora of highly efficient devices such as gas sensors, spintronic devices, nanoelectronics, and optoelectronic devices (Chakraborty and Chattaraj, 2017). An inorganic counterpart of graphene is the boron nitride doped system that can be functionalized with OLi_4_, CLi_6_, NLi_5_, BLi_7_, Al_12_Be to achieve some interesting properties (Chakraborty and Chattaraj, 2017). The M_3_O^+^ (M = Li, Na, K) functionalized graphene nanoflakes (Chakraborty and Chattaraj, 2016a) are known to sequestrate various polar molecules such as CO, NO, and CH_3_OH. Sequestration of gas molecules such as H_2_, O_2_, O_3_, CO, NO, and H_2_O through bare boron nitride flakes (BNF) and metal oxide, MO (M = Cu, Ag, Au) functionalized BNF are also reported (Chakraborty and Chattaraj, 2016b). In this review we report some optimization techniques for the generation of minimum energy structures of some selected clusters and also the bonding, reactivity, and different properties of some selected confined systems. Aromatic behavior and electride properties of some clusters are also investigated.

## Theoretical Background and Computational Details

Before optimizing the geometry of any system, we carefully ponder over the requirement of the study and select the level of theory maintaining a parity between the level of accuracy required and the computational cost to be incurred. Most often, the easiest way is to take into consideration the experimental data (if available) and select accordingly. The systems discussed in this article are optimized using the computational chemistry software package, Gaussian 09 (Frisch et al., 2009). We have used B3LYP (Lee et al., 1988; Becke, 1992), BP86 (Perdew, 1986a; Perdew, 1986b; Becke, 1988), *ω*b97X-D (Chai and Head-Gordon, 2008), PBE (Perdew et al., 1996; Perdew et al., 1997), TPSSTPSS (Staroverov and Scuseria, 2003; Tao et al., 2003), M06, M06-2X (Zhao and Truhlar, 2008), and M05-2X (Zhao et al., 2006) functionals for carrying out DFT calculations of various systems. The exact level of theory (method and basis set) chosen for the individual case studies is mentioned in the Results and Discussion section. Relativistic effects for heavier atoms are taken care of by effective core potentials (ECPs). The stationary states are better understood from the harmonic vibrational frequencies. The minimum energy structure and the transition state (TS) are identified with the presence of zero and one imaginary frequency, respectively.

The atomic charges, nature of interactions present within the systems, and the possible bond formation are analyzed with the help of natural population analysis (NPA) (Reed et al., 1985), Wiberg bond indices (WBI) (Wiberg, 1968) in the NBO scheme (Reed et al., 1988). The electron density topology is mapped using Bader’s quantum theory of atoms-in-molecules (QTAIM) (Bader, 1985) in Multiwfn (Lu and Chen, 2012). Parameters such as electron density [*ρ*(r_c_)], total electron energy density [*H*(r_c_)], local kinetic energy density [*G*(r_c_)], local potential energy density [*V*(r_c_)], and Laplacian of electron density [∇^2^ *ρ*(*r*)] are computed at the bond critical points (BCPs) and they help analyze the extent of covalent or ionic character present along that bond path. The NCI index reveals the localized binding interaction in a system, and the plot can be visualized as red, blue, or green regions in the NCIPLOT program (Contreras-García et al., 2011) depending on whether the interaction is repulsive, H-bond, or van der Waals, respectively. The nonlinear optical (NLO) properties are evaluated in terms of average linear polarizability ($\bar{\alpha }$), first (*β*) and second ($\gamma_{\|}$) hyperpolarizabilities.

ADF 2013.01 software (Baerends et al., 2013) is utilized to perform the energy decomposition analysis (EDA) (Morokuma, 1971) with the natural orbitals for chemical valence (NOCV) (Mitoraj et al., 2009). The interaction between two selected fragments of the studied system is represented in terms of three attractive and one repulsive energy terms. The attractive term includes electrostatic energy (Δ*E*_elstat_), orbital interaction energy (Δ*E*_orb_), dispersion interaction energy (Δ*E*_disp_), while the repulsive term is known as Pauli repulsion energy (Δ*E*_Pauli_).

$$\Delta E_{\mathrm{int}}=\Delta E_{\mathrm{elstat}}+\Delta E_{\mathrm{orb}}+\Delta E_{\mathrm{disp}}+\Delta E_{\mathrm{Pauli}}$$ (1)

In NOCV, the orbital term is represented as the sum of Δ*E* ^*k*^_orb_ (pairwise orbital energies) which is related to Δ*ρ* ^*k*^(r) (pairwise charge contributions).

$$\Delta E_{orb}=\sum_{k}\Delta E_{orb}^{k}$$ (2)

Atom-centered density matrix propagation (ADMP) (Iyengar et al., 2001; Schlegel et al., 2001; Schlegel et al., 2002) in Gaussian 09, and Born-Oppenheimer molecular dynamics (BOMD) in deMon2K software (Koster et al., 2011) are used to perform the dynamic study of the systems under discussion in this article.

For the global optimization study using PSO, ADMP-CNN-PSO, and FA, the algorithms are written in Python 3.7 programming language (Van Rossum and Drake, 2009). The single point energies (SPEs) are calculated using Gaussian 09 at the post-processing step. The calculations for all the clusters are performed using the B3LYP (Lee et al., 1988; Becke, 1992) functional of DFT. The basis set 6-311+G(d,p) (McLean and Chandler, 1980; Raghavachari et al., 1980) is used for the boron clusters, 6-311+G(d) for Al_4_^2−^, C_5_, and N_4_^2-^, 6-311G(d) for N_6_^4-^, and LANL2DZ (Dunning and Hay, 1977; Wadt and Hay, 1985; Hay and Wadt, 1985a; Hay and Wadt, 1985b) with ECPs for Au_*n*_ (*n* = 2–8) and Au_*n*_Ag_*m*_ (2 ≤ (*n+m*) ≤ 8). The algorithms are executed in a server with two Intel 2.70 GHz Xeon E5-2697 v2 processors (each with 12 cores and 30 threads) and a 256 GB RAM. The software Keras (Chollet, 2015) is used for interfacing with Python 3.7 with convolution neural networks (CNN).

## Results and Discussion

### Global Optimization Using Machine Learning Techniques

Minimization of a system’s energy functional is the most fundamental step in the determination of its ground state. Reaching the global minimum (GM) geometry, however, poses a number of challenges, the most important being the high probability of getting stuck in local minima in the PES. Swarm intelligence (SI)-based algorithms have turned out to be very effective in searching for optimal solutions in a given search space. Here we discuss three different techniques, PSO combined with DFT, PSO with CNN, and DFT-integrated FA. They do not need to implement any symmetry constraint, or consider bond characterization. DFT-PSO adjusts each particle’s trajectory at every time stamp while following the convergence criteria. We have successfully implemented these techniques to find the GM configurations for small-sized nonmetallic clusters such as Boron (B_5_ and B_6_) (Yuan et al., 2014), Carbon (C_5_) (Jana et al., 2019), and polynitrogen clusters (N_4_^2-^ and N_6_^4-^) (Mitra et al., 2021), and metallic clusters such as Al_4_^2-^ (Mitra et al., 2020), Au_*n*_ (*n* = 2–8), and Au_*n*_Ag_*m*_ (2 ≤ *n+m* ≤ 8) (Mitra et al., 2021).

#### PSO Combined With DFT (DFT-PSO)

**FIGURE 1:**
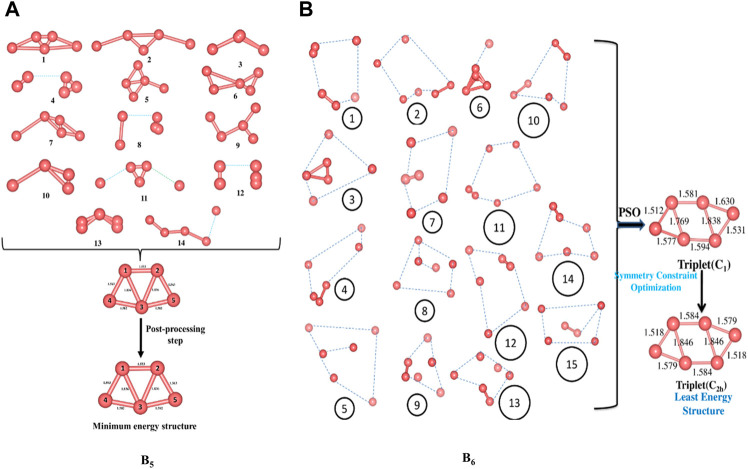
Randomly generated configurations of **(A)** B_5_ and **(B)** B_6_ and their convergence to their respective global minimum energy structures (Bond lengths are provided in Å). (Adapted with permission from Mitikiri et al., 2018. Copyright© 2021, John Wiley & Sons, Inc.).

We start off with 14 and 15 random structures for B_5_ and B_6_ (Figure 1), respectively, with velocity set at zero and maximum number of iterations set at 1,000. No significant change in bond length is detected after reaching the global best configuration at the end of the PSO run. Following this, the optimization is performed at the B3LYP/6-311+G(d,p) level as the post-processing step that helps align the symmetry of the PSO-obtained final structure and obtain the corresponding exact energy. The post-processing in this case takes only 20 s to complete, and the final geometry obtained is energetically very close (0.0015 eV difference) to that obtained at the end of the PSO run. The zero-point energy (ZPE) corrected energy, free energy, and enthalpy for B_5_ (*C*_2v_) are −123.9873, −124.0135, and −123.9821 a.u., respectively, while those for B_6_ (*C*_*2h*_) are −148.8100, −148.8381, and −148.8038 a.u., respectively. A comparison drawn between our method and other popular algorithms such as DFT-SA and DFT-BH reveals that while these two require a CPU time of 369.64 and 455.43 min to locate the minimum energy structure of B_5_, respectively, our method takes only 80.50 min. It is also observed that while the BH and the SA require 600 (unconverged) and 324 number of iterations, respectively, our modified PSO converges after only 138 number of iterations.

For the carbon clusters, 10 random configurations are chosen with initial velocity zero and 1,000 number of iterations. For the C_*n*_ (*n* = 3–6) clusters, linear geometries are obtained with *D*_*∞h*_ point group as the GM. For *n* = 4–6, a cyclic isomer for each of them is also obtained with point groups D_2*h*_, C_2*v*_, and *D*_3*h*_ for C_4_, C_5_, and C_6_, respectively, whereas for the relatively larger C_10_ cluster, the GM geometry is a *D*_10*h*_ ring structure. It is to be noted that the geometries and corresponding energies reported here match with those obtained from the experimental reports (Raghavachari and Binkley, 1987; Watts et al., 1992; Hutter and Lüthi, 1994; Pless et al., 1994; Martin and Taylor, 1996; Van Orden and Saykally, 1998). Again, comparing with DFT-SA and DFT-BH, we get encouraging results for our modified PSO approach. The total execution time for our technique is 143.30 min versus 215.98 and 5085.67 min for DFT-SA and DFT-BH, respectively.

**FIGURE 2:**
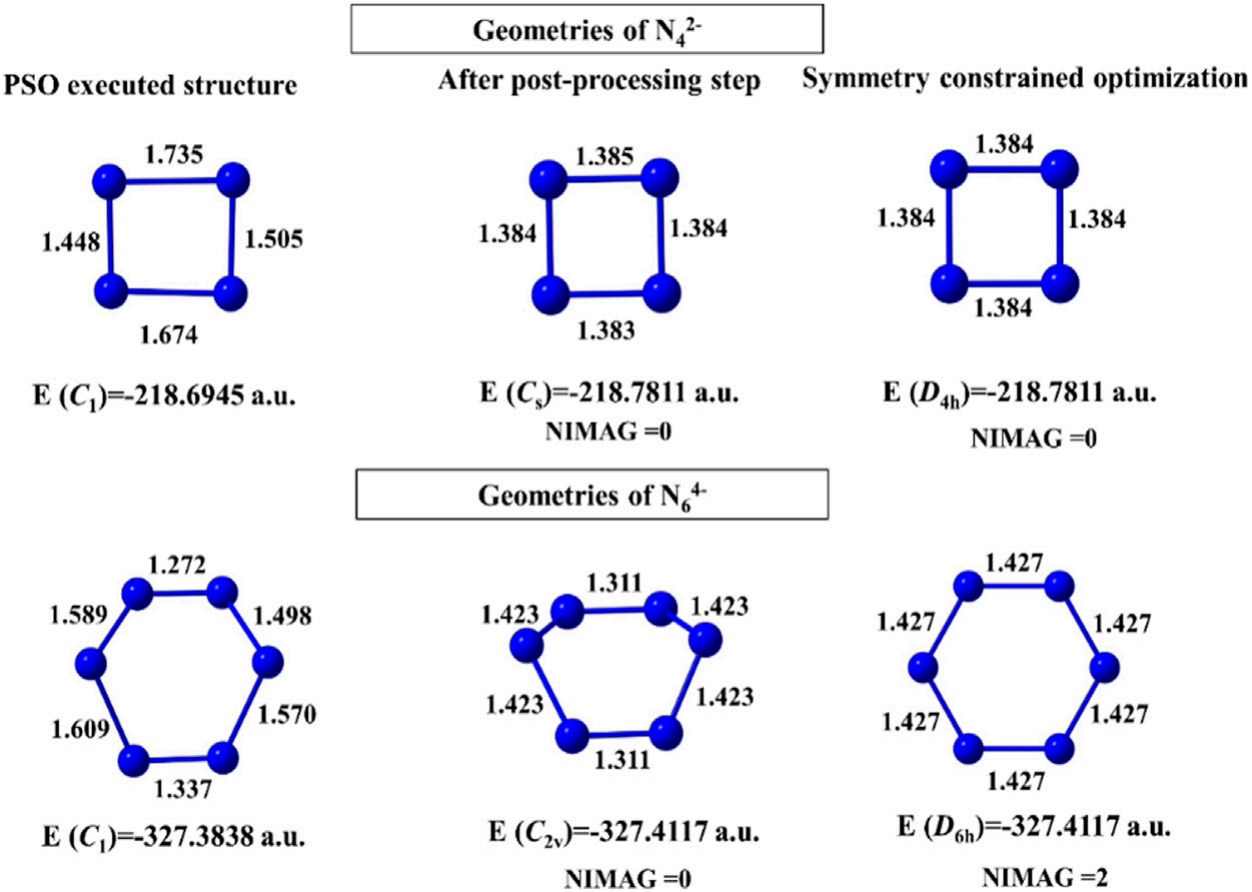
Structures of N_4_^2-^ and N_6_^4-^ clusters obtained at the end of the PSO run, post-processing step, and geometry-constrained optimization computed at the B3LYP/6-311 + G(d) level. (Reprinted from Mitra et al., 2021 with permission from Theoretical Chemistry Accounts, Springer Nature. Copyright© 2021, Springer-Verlag GmbH, DE.).

For N_4_^2-^ and N_6_^4-^ clusters, convergence takes place after 483 and 627 numbers of iterations with 228 and 323 min of execution time, respectively. The geometries obtained before and after the post-processing step are energetically very close (Figure 2). The N_6_^4-^ cluster, however, shows a higher order saddle point at the post-processing symmetry constrained optimization with point group *D*_6*h*_. In case of the binary gold–silver clusters (Au_*n*_Ag_*m*_), AuAg_2_ has a ring (*C*_*s*_) doublet GM, those with *n+m* = 5 have trapezoidal GM, *n+m* = 6, 7 have triangular 3D geometry (*C*_1_), and *n+m* = 8 failed to converge within the initial coordinates range of [−4, 4].

#### CNN With PSO (CNN-PSO)

**FIGURE 3:**
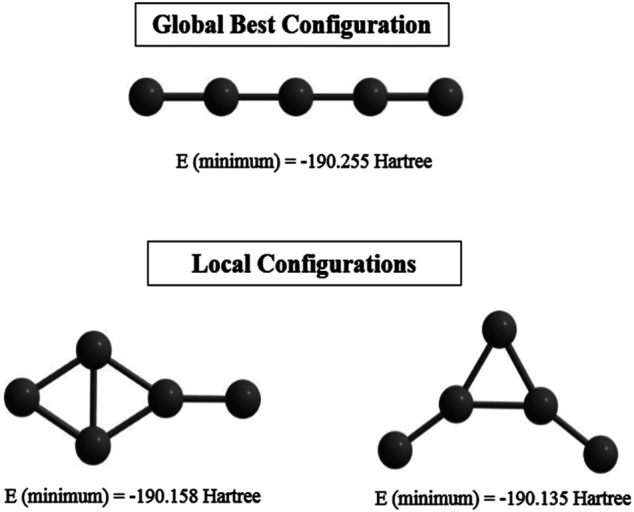
Global and local minimum energy structures of C_5_ cluster obtained at the end of the post-processing step computed at the B3LYP/6-311+ G(d,p) level. (Reprinted from Mitra et al., 2021 with permission from Theoretical Chemistry Accounts, Springer Nature. Copyright© 2021, Springer-Verlag GmbH, DE.).

Supervised learning (CNN) is performed on an initial guess set generated with the help of (ADMP) simulation. Their corresponding single point energies (SPEs) are calculated and stored to be read by the PSO to search for the GM geometry. Statistically relevant analysis is derived by making the method 8-fold (each with 30 files containing number of iterations and the SPEs). Remarkably high success rate (∼77–90%) is observed for this combined ADMP, CNN, PSO technique, indicating its efficiency in finding GMs. This technique is tested with the C_5_ cluster and we have obtained the previously reported (Van Orden and Saykally, 1998) linear geometry as the GM with a higher convergence rate. However, two local minima are also detected due to a premature convergence (Figure 3).

#### FA With DFT (DFT-FA)

**FIGURE 4:**
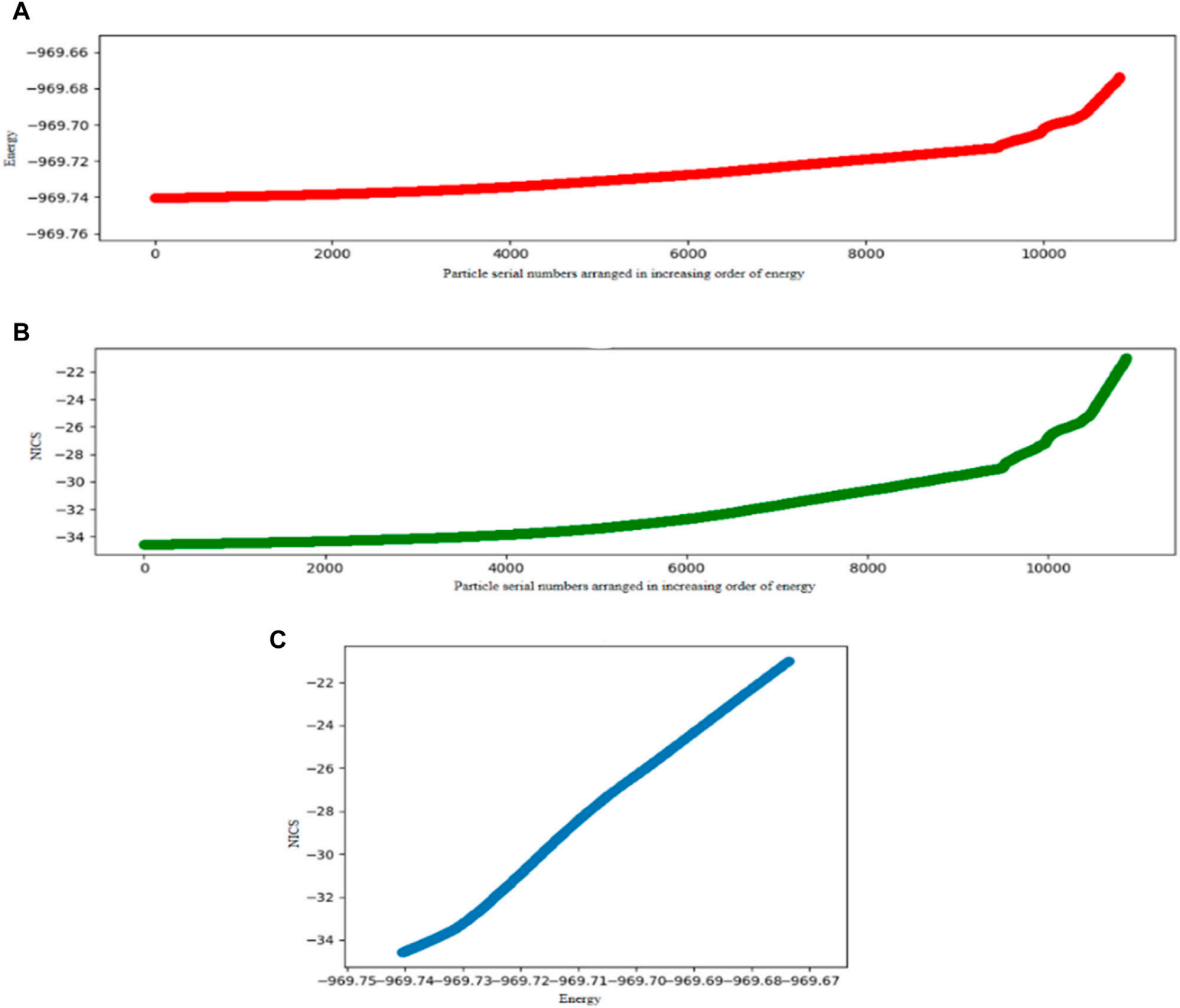
Energy profiles (in a.u.) and Nucleus-Independent Chemical Shift (NICS) values on Al_4_^2-^ where 4**(A)**, 4**(B),** and 4**(C)** represent plots of Energy vs. Particle serial numbers arranged in an increasing order of energy, NICS *vs*. Particle serial numbers arranged in an increasing order of energy and NICS vs. Energy, respectively. (Reprinted from Mitra et al., 2020 with permission from Theoretical Chemistry Accounts, Springer Nature. Copyright© 2020, Springer-Verlag GmbH Germany.).

A comparative study of the DFT-integrated FA algorithm with PSO is performed on Al_4_^2-^ cluster considering planar and nonplanar structures, and it turns out that the former performs better than the PSO. The mean convergence times for the PSO and FA are 68.22 and 61.25 min for the planar, and 85.13 and 74.40 min for the nonplanar approach, respectively. The corresponding success rates are also higher for the FA. Since we know that the GM of Al_4_^2-^ is planar, we have also investigated the search space of only the planar geometry to get a faster convergence since the number of variables decreases in this problem. Again, the modified FA performs better. A relation is also drawn between the stabilization energy of the system and the change in its aromaticity and number of iteration steps it takes to converge to the GM. The energy functional optimization and the NICS (0) value of Al_4_^2−^ are scanned and depicted in Figure 4. It is observed that the aromaticity increases with the decrease in the energy, *i.e*., with the increase in the stability of the system.

### Aromaticity of Clusters From a CDFT Perspective

It is well known from Hückel’s (4n+2) *π* electron theory (Hückel, 1931) and Pauling’s quantum mechanical description (Pauling and Sherman, 1933) that aromaticity of conjugated systems with cyclic and planar geometry is associated with their increased stability. Various structural, energetic, electronic, and magnetic behavior-based parameters are considered to analyze the aromaticity of the systems, among which the Nucleus-Independent Chemical Shift (NICS) (Schleyer et al., 1996) is, perhaps, the most widely used criterion for aromaticity. CDFT also plays an important role in aromaticity determination (Chattaraj et al., 2005; Chattaraj et al., 2006; Chattaraj et al., 2007), and it does so with the help of reactivity descriptors and associated electronic structure principles (Chattaraj et al., 1993; Chattaraj and Maiti, 2001; Pan et al., 2013b; Chakraborty and Chattaraj, 2021). The relative aromaticity index Δ*X*, where *X* could be energy (*E*), polarizability (*α*), electrophilicity (*ω*), or hardness (*η*), is defined as the difference between the respective indices in the cyclic and open (or localized) systems, *i.e*., Δ*X* = *X*_CYCLIC_ – *X*_OPEN(/LOCALIZED)_. They are observed to show a similar performance as those of the NICS and MCI values. They also provide valuable insights into the stability, reactivity, and electronic properties of the associated cluster.

All-metal aromatic Al_4_^2−^ (Li et al., 2001) and antiaromatic Al_4_^4−^ (Kuznetsov et al., 2003) clusters are investigated at the B3LYP/6-311G(d, p) level of theory to analyze their aromaticity from a CDFT perspective (Chattaraj et al., 2005; Chattaraj et al., 2006; Chattaraj et al., 2007). While Δ*η* > 0 represents aromaticity and Δ*η* < 0 represents antiaromaticity, the reverse is true in the cases of *E*, *α*, and *ω* indices. Their values for the aluminum clusters are compared with those of benzene (C_6_H_6_) and cyclobutadiene (C_4_H_4_). While Al_4_^2−^ and C_6_H_6_ show positive Δ*η* and negative Δ*E*, Δα, and Δ*ω* values indicating an aromatic behavior, C_4_H_4_ shows an exact opposite trend to account for its antiaromaticity. For the Al_4_^4−^ cluster, however, we have obtained somewhat contradictory results. Δ*E* and Δα values indicate the cluster’s antiaromatic nature, whereas Δ*η*, Δ*ω*, and NICS values reflect its aromatic nature. It is in conformity with the current knowledge that this cluster exhibits conflicting aromaticity. Experimental synthesis and theoretical studies reported along with ELF analysis (Li et al., 2001; Santos et al., 2005) suggest the cluster to be antiaromatic. Its σ-aromaticity directs the overall aromaticity by dominating over its π-counterpart as studied through NICS (Chen et al., 2003) and magnetic field induced current density (Havenith et al., 2004) analyses. Such conflicting aromaticity, along with other varieties of multiple aromaticity and antiaromaticity, δ- and Φ-aromaticity, bond stretch isomerism, *etc*. are exhibited by several other all-metal clusters (Zubarev et al., 2009; Zubarev and Boldyrev, 2011). Be_3_^2−^, Ca_3_^2−^, and Mg_3_^2−^ clusters are classified as aromatic in terms of the Δ*X* indices (Roy and Chattaraj, 2008; Giri et al., 2010). Other applications of aromatic clusters are studied by our group through molecular electronic transport (Khatua et al., 2008), hydrogen storage (Havenith et al., 2005; Giri et al., 2011a; Giri et al., 2011b; Das and Chattaraj, 2012; Srinivasu et al., 2012; Pan et al., 2012b), and Zn–Zn and Be–Be bond stabilization (Chattaraj et al., 2008; Roy and Chattaraj, 2008) in the domain of CDFT. The Δ*X* and NICS parameters show their versatility in quantifying the aromaticity of not just planar and cyclic systems, but also any other nonplanar closed structure. They are both easily computable and Δ*X* also has a conceptual lucidity since it originates from the electronic structure principles of CDFT.

### Structure, Bonding, and Reactivity of Various Molecular Electrides

Certain chemical entities contain loosely bound electrons not directly connected to any atom(s) within the cluster, but trapped within a hollow space (cavity of cage compounds or packing void in crystals) that act as anions. Such entities are known as electrides and they are known for their nonlinear optical (NLO) properties. Besides showing NLO properties, which is considered to be an identifiable character of an electride, it is also widely applicable in electron emission, catalysis, reversible hydrogen storage, super conductivity, *etc*. (Toda et al., 2007; Xu et al., 2007; Kitano et al., 2012). Organic electrides of crown ethers and cryptands (Ellaboudy et al., 1983; Ward et al., 1988; Dawes et al., 1991; Xie et al., 2000), and inorganic electrides such as [Ca_24_Al_28_O_64_]^4+^(4*e* ^−^) (Matsuishi et al., 2003), Y_5_Si_3_ (Lu et al., 2016), [Ba_2_N_2_](*e* ^−^), [Li_2_Ca_3_N_6_](2*e* ^−^) (Qu et al., 2019), [Ca_2_N]^+^(*e* ^−^) (Lee et al., 2013), [Y_2_C]^1.8+^·1.8*e* ^−^ (Zhang et al., 2014), Sr_5_P_3_ (Wang et al., 2017), and Yb_5_Sb_3_ (Lu et al., 2018) are well reported in the literature. A different class of electrides, known as molecular electrides, are basically guest@host complexes containing a significant amount of localized electron cloud within the void of the host. Cavity-containing molecular structures such as decaborane (Muhammad et al., 2009; Muhammad et al., 2011), pyrrole (Chen et al., 2005), tetracyanoquinodimethane (TCNQ) (Li et al., 2009), fullerene cages (Das et al., 2020), C_20_F_20_ (Wang et al., 2012), C_60_F_60_, extended (3.1.3.1) porphyrin (EP) (Saha and Chattaraj, 2018), and many more are utilized for this purpose. The guest atoms are usually alkali and alkaline earth metals. For a guest@host complex to be characterized as a molecular or cluster electride, certain criteria need to be fulfilled such as the presence of a non-nuclear attractor/maximum (NNA/NNM) (a non-nuclear critical point with a local maximum of electron density), a negative Laplacian of electron density [∇^2^ *ρ*(*r*_*c*_)], presence of an ELF basin near the NNM, high NLO properties, and a green region in the NCI plot showing accumulation of electron density.

The Mg_2_EP complex studied at the M06-2X-D3/6-311G(d,p) level of theory (Saha and Chattaraj, 2018) shows the presence of NNA in between the two Mg atoms where the value of ∇^2^ *ρ*(*r*_*c*_) < 0, with an ELF basin nearby. The electron population at said NNA is 1.02 *e* with 46% localization. The NLO properties in terms of $\bar{\alpha }$, *β*, and $\gamma_{\|}$ are calculated and compared with other electride systems that show that $\bar{\alpha }$ is higher while *β* and $\gamma_{\|}$ are lower in the studied systems. The donation of the loosely trapped electron to antibonding MOs of certain bonds in small molecules (H-H in H_2_, C-O in CO_2_, N-O in N_2_O, and C-H in CH_4_ and C_6_H_6_) results in the activation followed by the dissociation of the respective bonds (Saha and Chattaraj, 2018). Another study (Saha et al., 2019) performed by our group utilizes the modified form of *β*-diketiminate ligand (^Dipp^Nacnac) to hold four Mg atoms with two equivalent Mg(I)-Mg(I) bonds ([Mg_4_(LHDipp)_2_]^2–^) in its lower energy singlet state. Two NNAs are found to be present at the center of each Mg(I)-Mg(I) bond with an electron population of 1.18 |*e*| and 52% localization. The calculated values of $\bar{\alpha }$, *β*, and $\gamma_{\|}$ are 891.7, 0.0, and 8.9 × 10^5^ a.u., respectively, which clearly indicate the system to be classified as an electride. This system is further stabilized by sandwiching it between two K@crown-6-ether^+^ [K@CE]^+^ counter cations. Again, the C_60_ cage trapping a magnesium dimer (Mg_2_@C_60_) (Das et al., 2020) and a lithium trimer (Li_3_@C_60_) (Das and Chattaraj, 2021a) act as electrides as indicated by the presence of NNA at the center of the Mg-Mg bond path and Li_3_ cluster in the respective complexes. The Li_3_ cluster encapsulated within a B_40_ cage (Li_3_@B_40_) (Das and Chattaraj, 2020) shows a similar behavior with a lower electron population at the corresponding NNA owing to the electron deficiency in the B cage atoms. Binuclear sandwich complexes formed with Be and Mg dimers with C_5_H_5_^−^, N_5_^−^, P_5_^−^, and As_5_^−^ ligands forming M_2_(*η* ^5^-L)_2_ complexes (Das and Chattaraj, 2020) contain NNAs at the center of the M-M bonds with population varying between 0.95 and 1.39 and percentage localization ranging within 43–62%. Discernible substituent effects on electride characterizers have also been reported (Das and Chattaraj, 2021b).

### Noble Gas Encapsulated $B_{40}$ Cage

**FIGURE 5:**
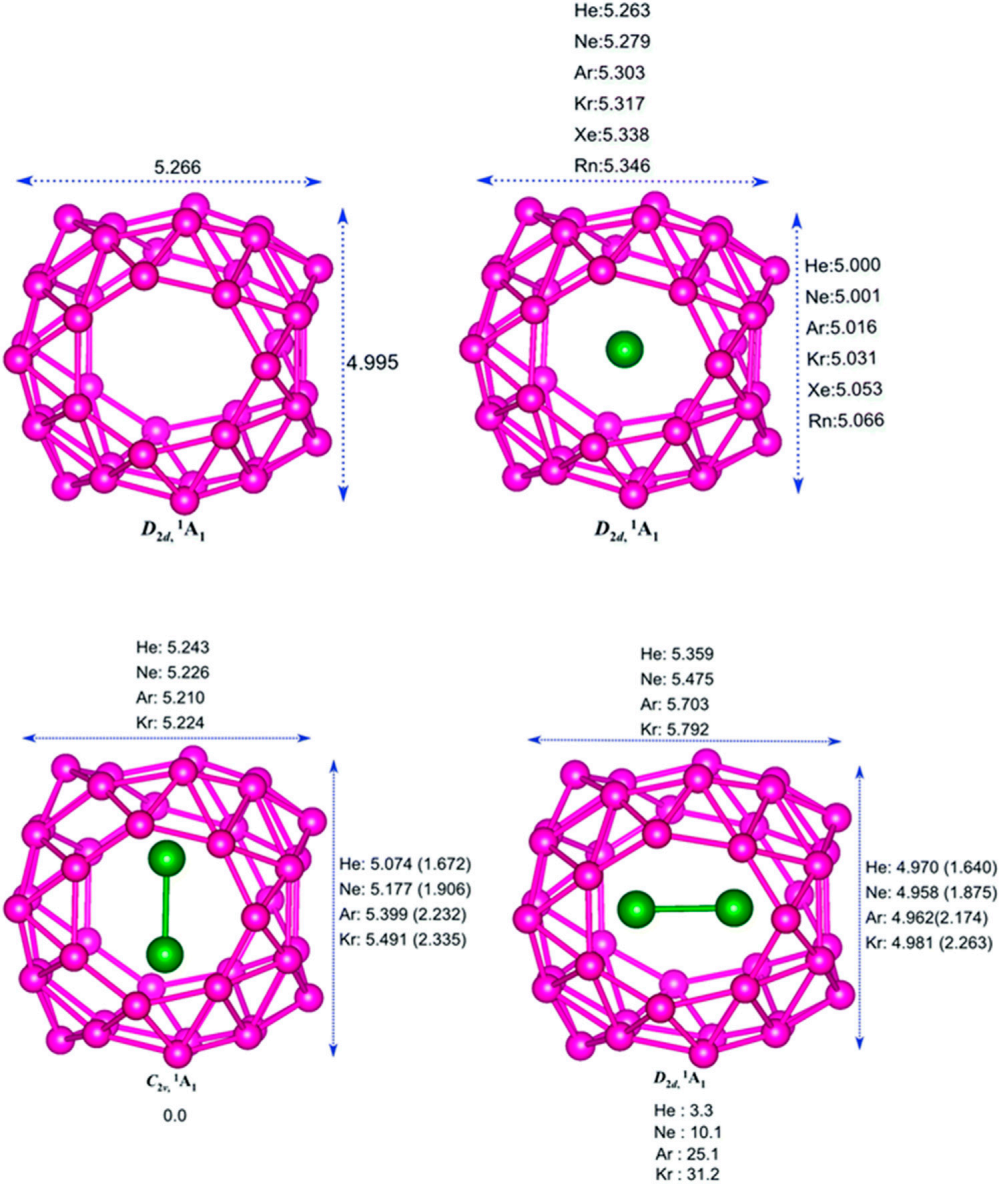
Optimized structures of B_40_, Ng@B_40_, *C*_2v_, and *D*_2d_ isomers of Ng_2_@B_40_ optimized at the *ω*B97X-D/def2-TZVP level. (Adapted from Pan et al., 2018 with permission from the PCCP Owner Societies.).

The encapsulation of noble gas (Ng) atoms in B_40_ cavitand along with their structure and interactions of Ng with the Ng and B atoms in the host–guest complex is discussed with the help of DFT-based computations (Pan et al., 2018). Dissociation energy (∆*E*_diss_) and Gibbs free energy change (∆*G*_diss_) are calculated to study the stability of the encapsulated complexes. NBO, EDA, and NOCV calculations have been done for studying the nature of bonding. The optimized structure of the B_40_ cage along with the Ng@B_40_ and Ng_2_@B_40_ systems (at *ω*B97X-D/def2-TZVP level) are depicted in Figure 5. The size of the B_40_ cage is suitable to accommodate He and Ne atoms at its center, whereas for heavier atom (Ar-Rn) encapsulation, the cavity diameter expands with the help of a certain amount of energy (preparation energy, Δ*E*_prep_). Although these complexes are thermochemically unstable, they remain in their encapsulated form on account of their high kinetic barrier. Despite that, the lighter Ng encapsulated complexes have very low ∆*E*_diss_ (−1.8 kcal mol^−1^ for He and −7.1 kcal mol^−1^ for Ne) as compared with the experimentally identified He@C_20_H_20_ complex (−33.8 kcal mol^−1^). For the heavier Ng atom-encapsulated Ng@B_40_ systems (Ng = Kr-Rn), the ∆*E*_diss_ increases with the size of Ng. The possibility of releasing Ng atoms in the dissociation process is either through B_7_ or B_6_ holes for the lighter He-Ar atoms, whereas the heavier ones can only escape through the B_7_ holes due to their larger size. Decapsulation through the B_7_ hole has Δ*G* ^≠^ values ranging within 84.7–206.3 kcal mol^−1^. The rate constant (*k*) calculated at 298 K for the dissociation through either the B_7_ or the B_6_ hole comes out to be pretty low suggesting that all the Ng@B_40_ systems are kinetically stable.

In the case of two Ng atoms encapsulation, the inter atomic distance decreases from that of their free state. For Ar_2_@B_40_ and Kr_2_@B_40_, larger repulsion results in the exergonic dissociation of Ng_2_@B_40_ into Ng and Ng@B_40_. Along with this, large ∆*E*_prep_ indicates the nonviability of Ar_2_B_40_ and Kr_2_B_40_. For even heavier Ng atoms (Xe and Rn), no minimum energy structures are obtained for the corresponding dimer encapsulation. The corresponding transition states of the Ng release process of Ng_2_@B_40_ (Ng = He-Ar) suggest that one Ng atom approaches and leaves through the B_7_ rings, while the other remains near the center of the cavity. The associated Δ*G* ^≠^ values for the He and Ne dimer encapsulated complexes are high enough for them to be kinetically viable.

The B_40_ cage exhibits a fluxional behavior due to the continuous interconversion between the B_6_ and B_7_ rings caused by the transfer of one B center from B_7_ to B_6_. The encapsulated system, Ng@B_40_, also shows similar dynamic behavior as that of the bare B_40_. The Ng atom inside the cage does not have any substantial influence on the fluxionality of the cage which is reflected in the free energy barrier values (16.4 kcal mol^−1^ for the bare cage, and a range of 16–18.9 kcal mol^−1^ for Ng@B_40_). Topological analysis of Ng@B_40_ explains the nature of the interactions therein. ∇^2^ *ρ*(r_c_) and *H*(r_c_) values being positive at the BCPs of Ng-B bond suggest the presence of noncovalent character in most of the complexes except for Rn@B_40_, Ar_2_B_40_, and Kr_2_B_40_, where *H*(r_c_) < 0. For the encapsulation of Ng_2_ (Ng = Ar_2_ & Kr_2_) the Ng-Ng bond becomes partially covalent in contrast to their noncovalent character in the free state, except in the cases of He_2_@B_40_ and Ne_2_@B_40_ where no covalency is imparted. From NBO analysis it has been shown that Ng → B_40_ charge transfer increases with increasing the size of the Ng atoms. The electron transfer further increases in the case of Ng_2_ encapsulation. Along He–Rn, an increase in WBI values indicates that the increasing size of Ng atoms increases the degree of covalency between the Ng and B centers. EDA analysis reveals a high positive ∆*E*_pauli_ which leads to positive ∆*E*_int_ suggesting the interaction to be repulsive in case of the heavier noble gas encapsulated B_40_ systems. Also, both the attractive terms, ∆*E*_elstat_ and ∆*E*_orb_, increase with the increasing size of Ng atoms.

### Small Gas Molecule Encapsulation Within Octa Acid Cavitand

**FIGURE 6:**
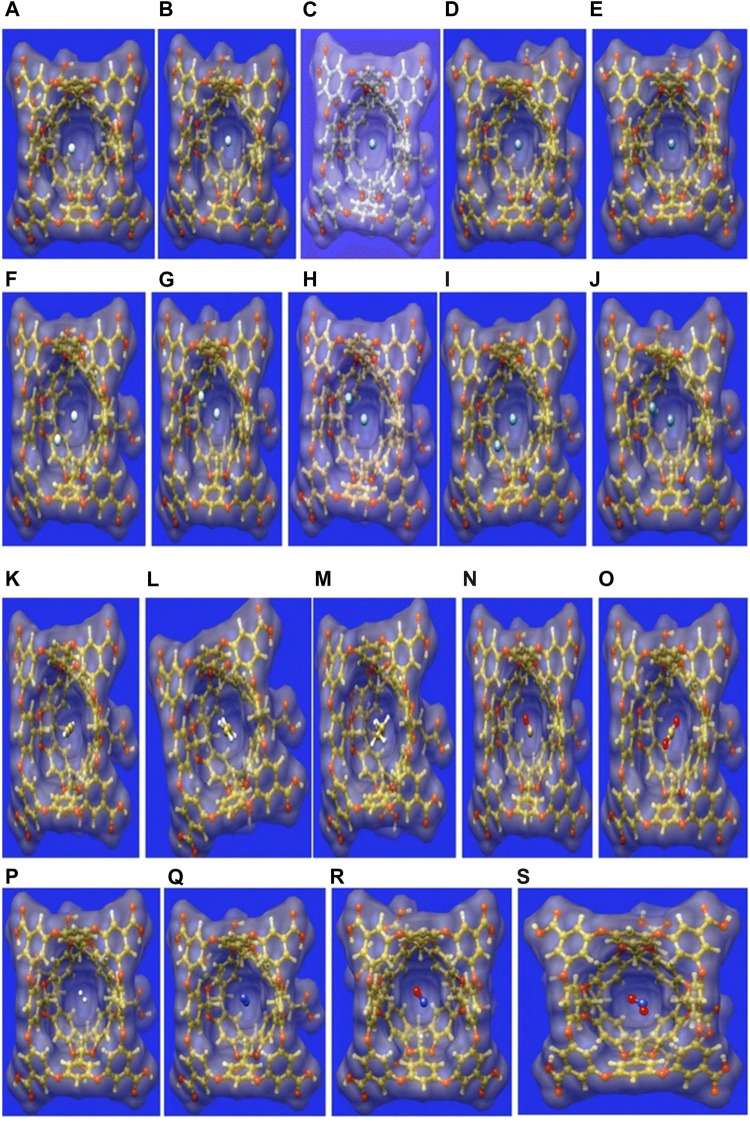
The surface representation of the optimized geometries of the guest encapsulated OA, where the guests are: **(A)** He, **(B)** Ne, **(C)** Ar, **(D)** Kr, **(E)** Xe, **(F)** He_2_, **(G)** Ne_2_, **(I)** Ar_2_, **(J)** Kr_2_, **(K)** Xe_2_, **(L)** C_2_H_2_, **(M)** C_2_H_4_, **(N)** C_2_H_6_, **(O)** CO, **(P)** CO_2_, **(Q)** H_2_, **(R)** N_2_, **(S)** NO, and **(T)** NO_2_. (Adapted from Chakraborty et al., 2016 with permission from Theoretical Chemistry Accounts, Springer Nature. Copyright© 2016, Springer-Verlag Berlin Heidelberg.).

Small gas molecules such as C_2_H_2_, C_2_H_4_, C_2_H_6_, CO, CO_2_, NO_2_, NO, N_2_, H_2_, and Ng atoms (He_*n*_-Xe_*n*_, *n* = 1,2) are selected as guest molecules encapsulated in OA cavitand (Figure 6) and analyzed via DFT approach (Chakraborty et al., 2016). The systems under study are optimized at the *ω*B97X-D/6-311G(*d*,*p*) level of theory (LanL2DZ basis set with ECP is used for Xe). There are two possible cavities for the accommodation of the guest atoms, the inner cavity of OA is more suitable as it increases the host–guest interaction. In the case of Ng@OA, due to encapsulation of Ng atoms, no notable distortion is observed in the OA. For Ng_2_@OA systems, one guest atom can occupy the center of the host cavity, whereas the other one remains in the outer cavity. The Ng–Ng bond distances inside OA are 3.6, 3.7, 3.8, 3.9, and 4.1 Ǻ, respectively, for He_2_, Ne_2_, Ar_2_, Kr_2_, and Xe_2_. In the cases of CO@OA and NO@OA, the guest molecules prefer to stay in the inner cavity of OA. Their orientation with respect to the two nearest benzene-like fragments is almost perpendicular, whereas it is parallel to the rest of the benzene fragments of OA. The presence of these π electron clouds close to the encapsulated guests is expected to have a significant impact on the stability of the complexes, the nature of interaction, and dynamical behavior as well. For N_2_@OA and H_2_@OA, N_2_ and H_2_ remain well inside the inner cavity. Thermochemical study reveals that except for He@OA, in all cases, *D*_0_ value is positive indicating the stability of the host–guest complexes concerning their dissociation into the corresponding individual components. Going from lighter to heavier Ng atoms (also for Ng_2_@OA), *D*_0_ value increases, *i.e*., the host–guest interaction increases. This could most likely be due to the increasing polarizability of the Ng atoms down the group. The dissociation channels for all the encapsulated complexes have positive *D*_0_ values. Most of them, however, dissociate spontaneously at room temperature except in the cases of Kr, Kr_2_, Xe, C_2_H_2_, C_2_H_4_, C_2_H_6_, and N_2_ guest molecules. Thus, the encapsulation of the mentioned guest molecules is favorable at 298 K. In the cases of NO/NO_2_@OA and CO/CO_2_@OA, an increase in *D*_0_ value is observed from NO to NO_2_ and from CO to CO_2_. It can thus be deduced that the encapsulation of NO_2_ and CO_2_ inside OA forms more stable complexes compared with NO and CO, respectively. Having said that, it is to be noted that all the four complexes have favorable dissociation channels at room temperature. The hydrocarbons have better interaction with the OA and hence are not prone to dissociation at ambient temperatures.

The interaction between the host and guest moieties is analyzed with the help of NBO, NCI, and EDA. All the Ng atoms acquire some positive charges, *i.e*., transfer of electron density occurs from the Ng atoms to the host OA surface. The donation primarily occurs from the lone pair (LP) of Ng to the C-H antibonding orbital of OA for all the Ng atoms [except Ne where it takes place from LP to antibonding Rydberg state (Ry*)] and for CO, NO, N_2_, and CO_2_ molecules. WBI values for Ng–Ng and Ng–OA interactions indicate a purely noncovalent character therein. From this discussion it is clear that the confinement brings about an increase in the reactivity of all the guest atoms/molecules within the OA. NCI isosurfaces show the presence of green surface around the guest molecules indicating van der Waals interaction that stabilizes the host–guest complexes. EDA results show that there exists closed shell type of interaction between guest Ng/H_2_ and OA. The Δ*E*_disp_ and Δ*E*_orb_ are the largest and the smallest contributors toward the total attractive interaction, respectively. The former increases in magnitude with increasing size of the encapsulated Ng atoms. For the guest hydrocarbons, the contribution from ∆*E*_disp_ increases and Δ*E*_elstat_ decreases with the increasing number of H atoms. This is because the molecules such as C_2_H_2_ and C_2_H_4_ containing labile electron cloud can accumulate enough positive charge to favorably interact with the electron-rich fragments of the OA. This makes the contribution from ∆*E*_elstat_ very important in stabilizing these complexes. The contribution from charge transfer and polarization are very less toward ∆*E*_tot_ as indicated by the very low values of Δ*E*_orb_. A similar type of situation is observed for CO/CO_2_@OA complexes. For nitrogen-containing guests, the ∆*E*_orb_ outweighs the ∆*E*_elstat_ contribution toward Δ*E*_tot_. Since the main stabilizing factor for all the complexes is ∆*E*_disp_, the nitrogen-containing guest molecules are prone to be affected by the polarization or charge transfer by OA, in comparison with the other guest molecules. ADMP simulation performed at 298 K shows that all the Ng atoms (except Ne) are prone to leaving the cavity, whereas at 50 K they remain within the OA. Polar molecules such as CO, CO_2_, NO, and NO_2_ have a higher tendency to stay within the OA than the nonpolar H_2_. Again, C_2_H_2_, C_2_H_4_, and N_2_ containing π electron cloud also prefer to stay inside OA. From the above thermochemical, kinetic, and dynamical analyses, it can be said that OA makes a reasonably good choice for accommodating gas molecules.

### Cucurbit[*n*]urils as a Host Moiety

Cucurbiturils are methylene-linked macrocyclic molecules having glycoluril unit [=C_4_H_2_N_4_O_2_=] as a building block. This repeating glycoluril unit can bind with hydrogen with sufficient amount of binding energy. Thus CB[*n*] can be designed as an effective hydrogen storage compound. The nitrogen and the oxygen centers are found to be the most active centers to bind with hydrogen with positive binding energy. It was found that (CH_3_)_2_C_4_H_2_N_4_O_2_(CH_3_)_2_ unit can interact with total 13H_2_ atoms (Pan et al., 2013a). Since hydrogen has an electric quadrupole moment, a charge–quadrupole interaction plays a pivotal role in binding the hydrogen with the host. NPA charge analysis reveals that the charge transfer occurs from the N and O centers to the σ* orbital of $H_{2}$ molecule.

Among the CB[*n*] family, CB[7] can accommodate five H_2_ molecules endohedrally. The O centers can adsorb a total of 28 $H_{2}$ molecules (two per O atom) and 19 H_2_ molecules get adsorbed at the N centers exohedrally, making it a total of 52 hydrogen molecules. The binding energy and adsorption enthalpy are positive and negative, respectively, indicating CB[7] to be a potentially promising H_2_ storage material. The gravimetric wt% of hydrogen for the CB[7] adsorbing 52 H_2_ molecules comes out to be 8.3 with an average binding energy of 7.8 kJ mol^−1^. These values are very encouraging when compared with various other potential hydrogen storage materials such as α-cyclodextrin (Zhu et al., 2010), COFs (Klontzas et al., 2008; Li et al., 2010), MOFs (Bhatia and Myers, 2006; Vitillo et al., 2008), Li-doped nanotubes (Wu et al., 2008), and polyacetylenes (Li and Jena, 2008), with average binding energies ranging within 5–8 kJ mol^−1^.

**FIGURE 7:**
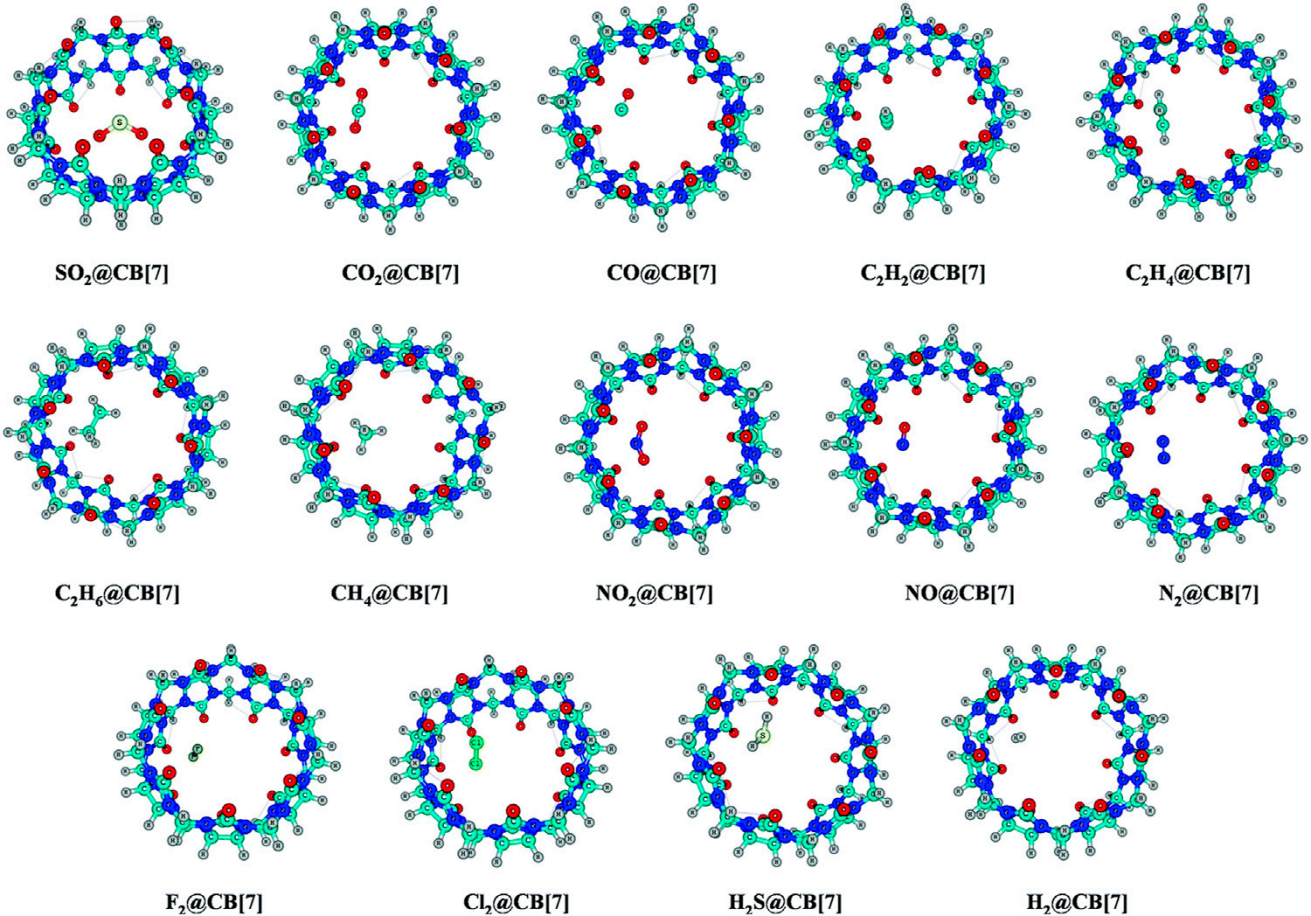
Optimized geometries of the guest encapsulated CB[7] systems at the ωB97X-D/6-311+G(d,p) level of theory. (Reproduced from Pan et al., 2017 with permission from the PCCP Owner Societies.).

The endohedral adsorption of gas molecules such as C_2_H_2_, C_2_H_4_, C_2_H_6_, CH_4_, CO, CO_2_, NO_2_, NO, N_2_, H_2_, F_2_, Cl_2_, H_2_S, and SO_2_ into the CB[7] cavitand is depicted in Figure 7. Geometry optimizations are performed at the ωB97X-D/6-31G(d,p) and ωB97XD/6-311+G(d,p) levels (Pan et al., 2017). Both the CB[7] cage and the encapsulated gas molecules remain unaffected by the encapsulation. For SO_2_ encapsulation, the binding enthalpy shows the highest value (14.3 kcal mol^−1^) followed by Cl_2_ and C_2_H_2_ (11.6 and 10.4 kcal mol^−1^, respectively). CB[7] also encapsulates C_2_H_4_ and C_2_H_6_ more favorably than CO_2_, NO_2_, and H_2_S. The binding enthalpies for NO/F_2_/N_2_/CO/CH_4_@CB[7] systems vary from 4.7 to 5.8 kcal mol^−1^, the highest value corresponding to CH_4_. From the enthalpy values it is clear that CB[7] can selectively adsorb SO_2_ among various gas molecules, and hence can be applicable in the SO_2_ separation process from gas mixtures. ∆*G* value suggests that C_2_H_6_ is less prone to be encapsulated than CO_2_ inside CB[7]. SO_2_, Cl_2_, and C_2_H_2_ adsorb with negative Δ*G* values, whereas those of C_2_H_4_ and CO_2_ are slightly endergonic (0.6–0.7 kcal mol^−1^). The corresponding Δ*G* values for the adsorption of C_2_H_6_, N_2_, F_2_, NO_2_, NO, and H_2_S vary within 1.3–2.8 kcal mol^−1^ at 298 K temperature. EDA results show that for all the discussed complexes, ∆*E*_disp_ contributes more toward the stabilization of the host–guest systems. ∆*E*_elstat_ term also plays an important role here. In the hydrocarbons, as the number of H atoms increases, ∆*E*_elstat_ decreases gradually due to the reduction in the acidic character of the H atoms. Higher ∆*E*_elstat_ and lower ∆*E*_pauli_ values in the SO_2_ encapsulated complex make the interaction between the host and the guest stronger than for C_2_H_4_ and C_2_H_6_ analogues. This again validates the higher SO_2_ selectivity of CB[7].

**FIGURE 8:**
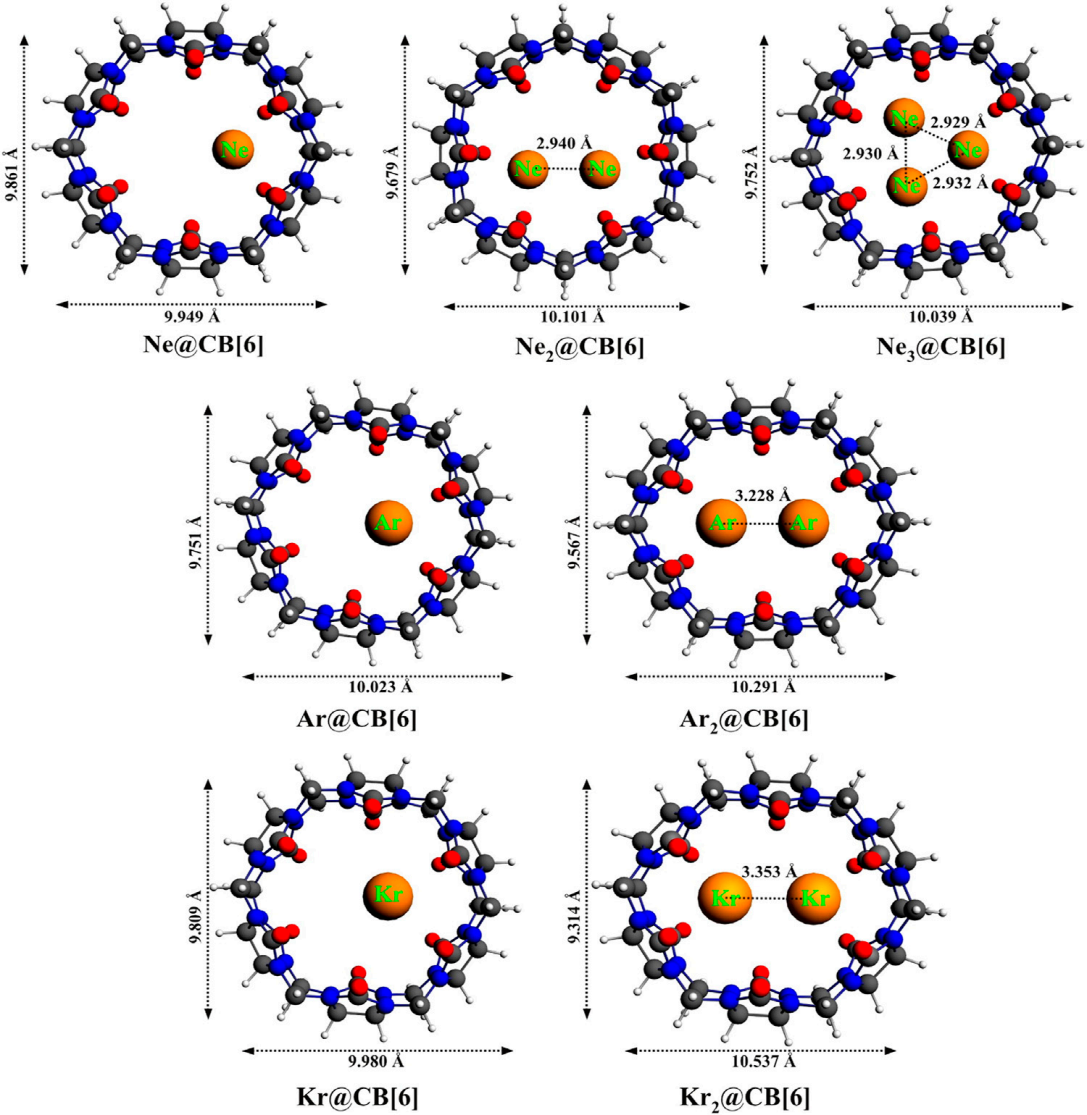
Optimized geometries of noble gas encapsulated CB[6] complexes at the ωB97X-D/6-311G(2d,p) level. (Reprinted with permission from Pan et al., 2015. Copyright© 2015, American Chemical Society.).

Cucurbit[6]uril, among the CB[*n*] family, is found to be a compatible host for the encapsulation of Ngs within its cavity (Pan et al., 2015). The optimized geometries at the *ω*B97X-D/6-311G(2*d*,*p*) level of theory of the Ng_*n*_@CB[6] complexes are provided in Figure 8. CB[6] effectively accommodates three Ne atoms, but can only trap two of the large Ar and Kr atoms. No significant distortion in the cage is observed for trapping all the three Ne atoms or for the first atom of Ar and Kr, whereas inserting a second atom deforms the shape of the host. The Ng dissociation process becomes more endothermic as we move from Ne to Kr. At 298 K, the dissociations of all Ng_*n*_@CB[6] are exergonic except Kr@CB[6]. At 77 K, apart from the second Ng (Ar and Kr) atom dissociation from Ng_2_ encapsulated CB[6], all dissociations become endergonic. Kr encapsulation at 298 K and 1 atm pressure is thermochemically favorable, whereas for Ne and Ar encapsulation, high pressure and moderately low temperature are preferred.

**FIGURE 9:**
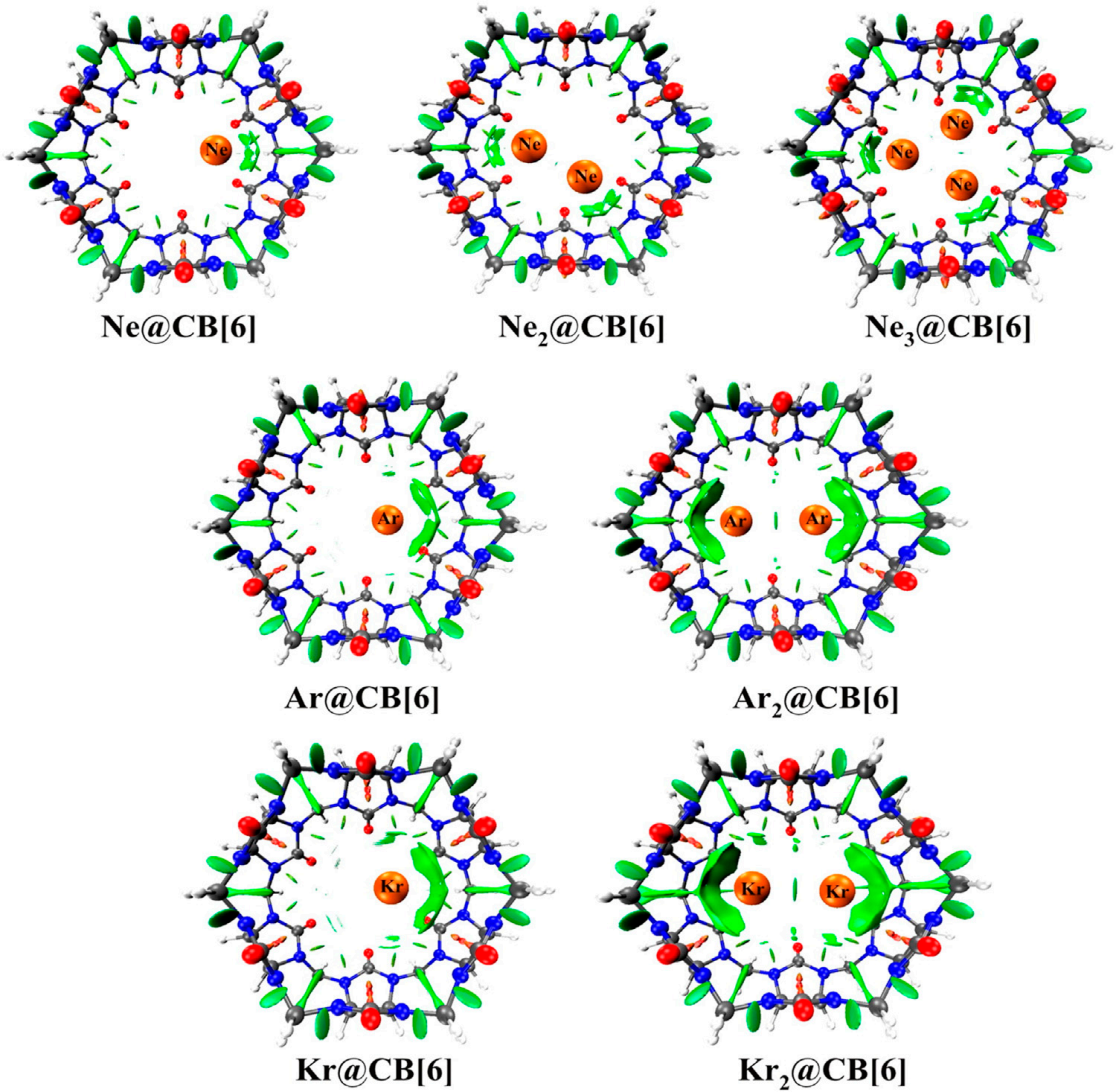
NCI plots of Ng_*n*_@CB[6] complexes. (Reprinted with permission from Pan et al., 2015. Copyright© 2015, American Chemical Society.).

NPA charge analysis reveals N and O to be negatively charged in Ng_*n*_@CB[6], while C and H have positive charges. Slight charge transfer (∼0.01 *e* ^*-*^) occurs from Ng→CB[6] moiety. Small *ρ*(r_c_) value and positive ∇^2^ *ρ*(r_c_) and *H*(r_c_) values from topological analysis suggest the interaction to be of closed shell type. ELF analysis shows an absence of electron localization between the Ng-Ng and Ng-cage atoms, corroborating the result obtained from AIM. EDA analysis reveals the contribution from ∆*E*_disp_ to be the largest, followed by ∆*E*_elstat_, and the smallest contribution is from ∆*E*_orb_, all of which gradually increases going from Ne to Kr. The green surfaces observed between the Ng and CB[6] units in the NCI isosurface (Figure 9) are an indication of a small van der Waals interaction, which increases with the size of the Ng atoms. The dynamical study (*ab initio* MD) for 1 ps and at 298 K reveals that Ne and Ar remain inside the cavity, whereas Kr and all the Ng_2_ in Ng_2_@CB[6] move toward the open end but do not leave the cage. At 77 K, all guests stay inside the host.

### Small Molecules Bound Metal Coordinated Boron Cluster

**FIGURE 10:**
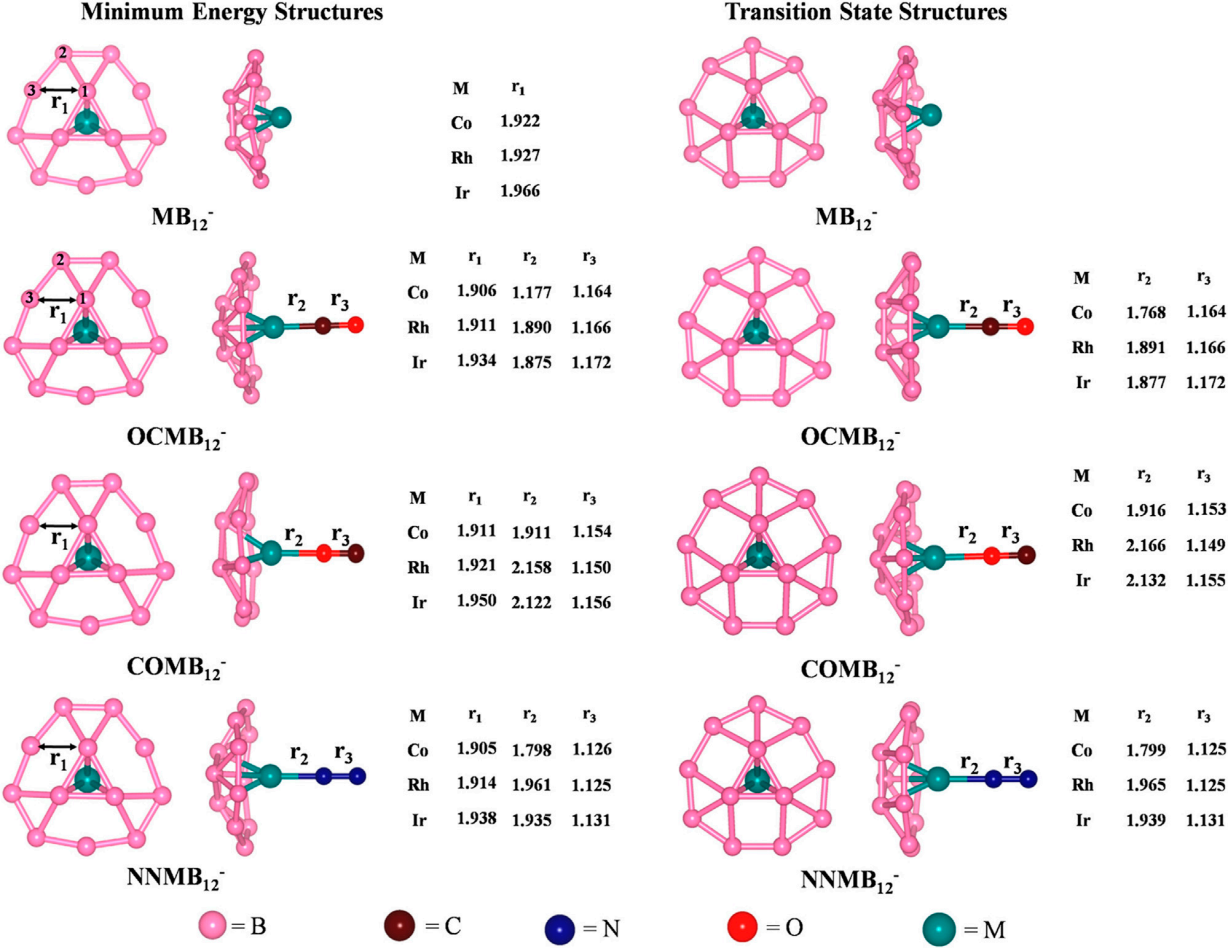
Optimized geometries of MB_12_^-^ and ligand bound MB_12_^-^ at the PBE/def2-TZVPPD level, and their transition state structures for the internal rotation of the B_3_ ring. Bond distances are provided in Angstrom unit. (Reprinted with permission from Saha et al., 2017. Copyright© 2017, American Chemical Society.).

The activation of small molecules by a metal-supported boron cluster is studied through DFT calculations (Saha et al., 2017). They are known to be applicable in nanomaterial building blocks, automobiles (Norbye, 1971; Jiménez‐Halla et al., 2010) *etc*. The global minimum energy structures calculated at PBE/def2-TZVPPD (Perdew et al., 1996; Weigend and Ahlrichs, 2005) level of MB_12_^-^, CO@MB_12_^-^, N_2_@MB_12_^-^ clusters and their corresponding TSs for the internal rotation of the B_3_ ring are provided in Figure 10. The coordination of the small molecules with the MB_12_^-^ cluster forms an umbrella-shaped structure in which the M-L bonds act like the stick of the umbrella. The coordination of CO with the metal center can take place through both the C and O ends, the former producing a more stable isomer. For both OCMB_12_^_^ and NNMB_12_^_^, the Ir-L bond has the highest strength, followed by Co and Rh, while for a particular M center, CO forms a stronger bond than N_2_. ∆*G* values of these complexes are highly positive which suggest that the corresponding complexes are thermodynamically stable concerning the dissociation process. The O-side bound isomers, however, have low positive ∆*G* for Co and Ir, and become slightly negative for Rh. They can be made viable by lowering the temperature. The N−N and C–O bonds get lengthened due to complexation in the order COMB_12_^-^ < N_2_MB_12_^-^ < OCMB_12_^-^ causing a red shift in their bond stretching frequencies which is the highest for the Ir analogues.

**FIGURE 11:**
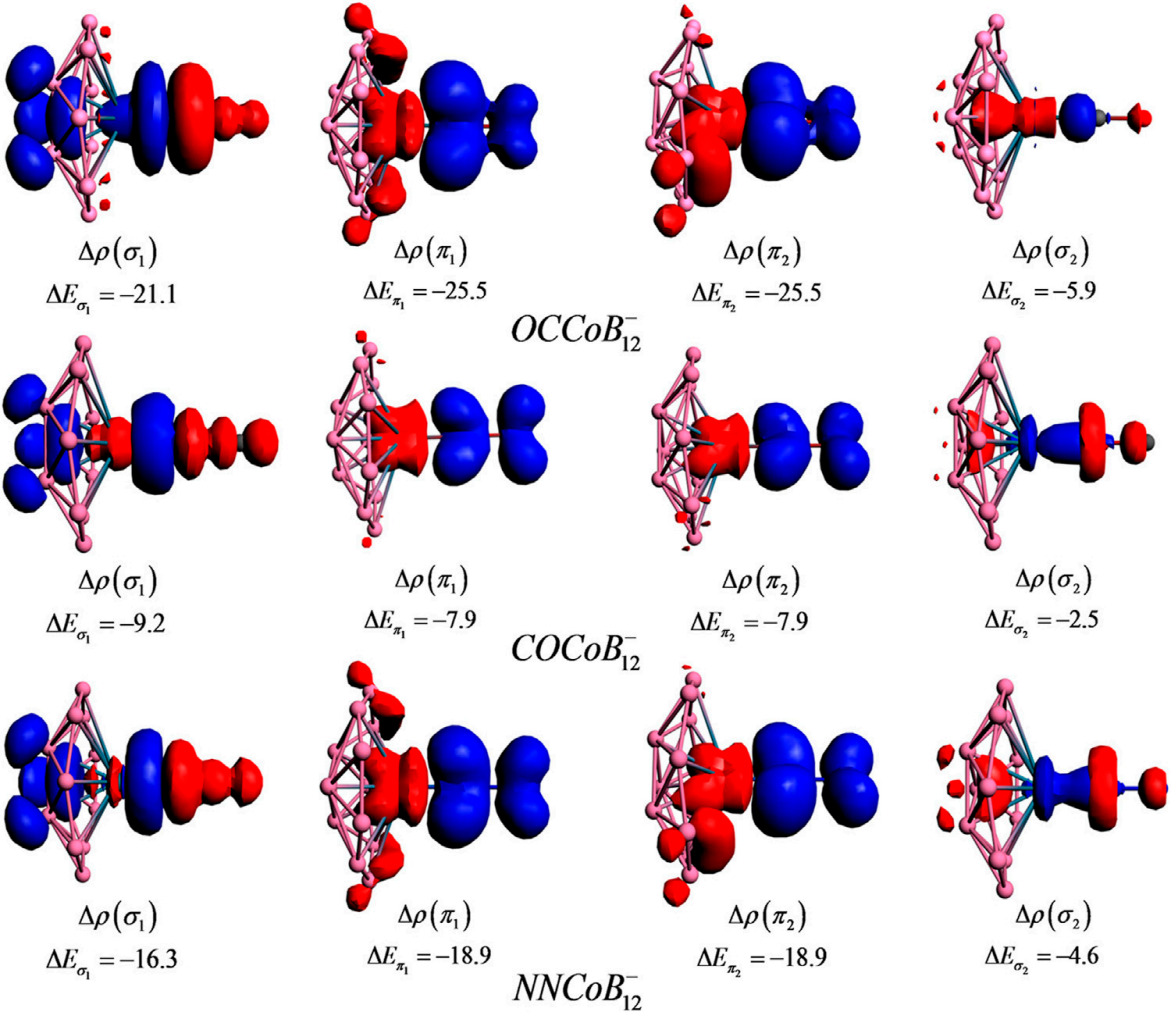
Deformation density plots of the pairwise orbital interactions in LCoB_12_^-^ (OC, CO, and NN) systems at the revPBED3/TZ2P//PBE/def2-TZVPPD level. Energies are provided in kcal/mol. (Reprinted with permission from Saha et al., 2017. Copyright© 2017, American Chemical Society.).

NBO analysis reveals that upon complexation, the metal centers get more negatively charged, apart from COIrB_12_^_^ and NNIrB_12_^_^, where Ir still contains positive charge (less than that in IrB_12_^−^). L→M and M→L back transfers take place, and in certain complexes the latter completely compensates (or overcompensates) the former which is indicated by the zero (or negative) charge on the ligand. The Wiberg bond indices suggest that the covalent character follows the order M-C in OCMB_12_^-^ > M-N in N_2_MB_12_^-^ > M-C in COMB_12_^-^. From EDA-NOCV, it is seen that the bonding between the metal and the ligand is predominantly orbital and electrostatic interactions (in more or less equal contributions), indicating the L-M bonds to have both covalent and ionic characters. For OCMB_12_^-^, however, the contribution from ∆*E*_elstat_ is higher than ∆*E*_orb_. Figure 11 shows the deformation densities [Δ*ρ*(r)] for the pairwise orbital interactions for the LMB_12_^_^ complexes, where a shift in the electron density occurs from the red to the blue region. The Δ*ρ*($\sigma_{1}$) plot reveals that the shift of electron density occurs through the L→M→B scheme. Δ*ρ*(π_1_) and Δ*ρ*(π_2_) explain the π electron density shift from the *d*_L→M_. The extent of L←M π-back-donation is greater than the L→M *σ*-donation, causing a red-shift in its stretching frequency. The *σ*-donation occurs from the HOMO_(CO)_ to the $\mathrm{LUM}O_{\left(\mathrm{LM}B_{12}^{-}\right)}$ fragment and *π*-back-donations occur from the degenerate $\mathrm{HOM}O_{\left(MB_{12}^{-}\right)}$ to the degenerate π* LUMO_(CO)_.

An internal rotation of the inner B_3_ ring with respect to the outer B_9_ ring occurs within the MB_12_^-^ cluster. The energy barrier associated with this rotation is reported (Popov et al., 2014; Liu et al., 2016) to follow the order Co > Rh > Ir. BOMD simulation at 800 K shows the L-M bonds to be intact during the rotation. This makes the complex seem like a spinning umbrella with the L-M bond as the stick.

### Hydrogen Storage in Clathrate Hydrates, Li-Doped Clusters, and Super Alkalis

**FIGURE 12:**
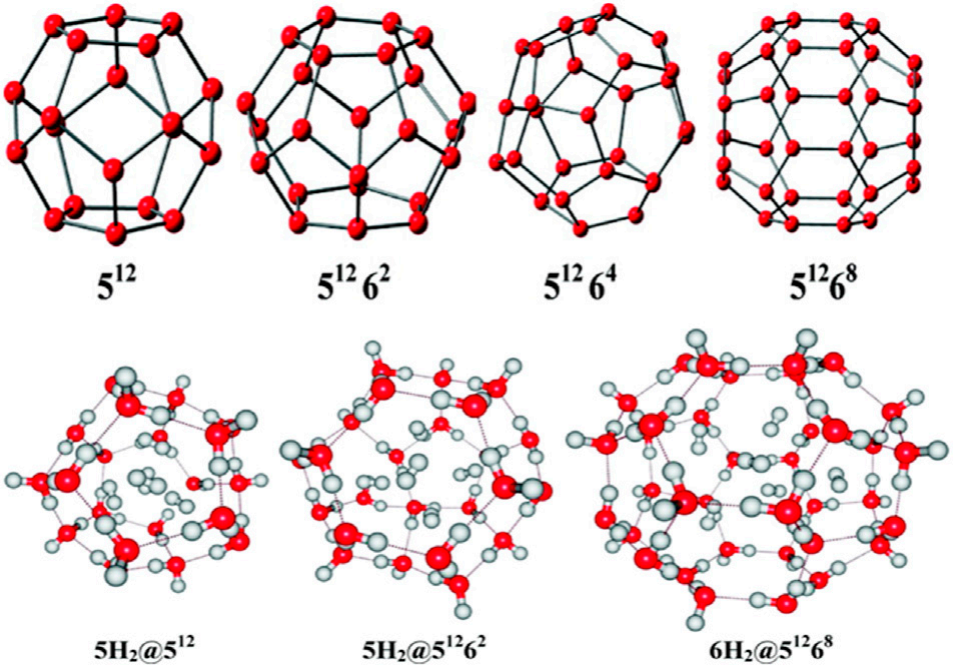
Optimized geometries of the clathrate hydrates along with their maximum possible H_2_ molecule encapsulated complexes. (Adapted with permission from Chattaraj et al., 2011. Copyright© 2011, American Chemical Society.).

Clathrate hydrates, a class of inclusion compounds, are known to encapsulate guest compounds within its hydrogen bonded polyhedral cage (Mao et al., 2002; Mao and Mao, 2004; Lee et al., 2010). They constitute a very effective host for hydrogen storage. Four types of clathrate hydrates and their maximum possible hydrogen-encapsulated complexes studied at B3LYP/6-31G(d) level (Chattaraj et al., 2011) are depicted in Figure 12. 5^12^ represents the cavity having 12 pentagonal faces, whereas 5^12^6^*k*^ (*k* = 2,4,8) represents 12 pentagonal faces along with *k* hexagonal faces. Here we discuss the structure, bonding, and stability of the bare and hydrogen-encapsulated complexes from a density functional theory perspective. For *n*H_2_@5^12^ complexes it is seen that for the first H_2_ encapsulation, the process is energetically favorable although the overall nH_2_ encapsulation is method dependent. Owing to the small size of 5^12^ cavity, it can accommodate a maximum of five H_2_ molecules, after which a deformation in the cavity is observed. The GM for H_2_ confinement in the 5^12^ cavity occurs endohedrally. The H_2_ molecules favor the inside of 5^12^ more than the outside. In the case of 5^12^6^2^ cage, it can also take up a maximum of five H_2_ molecules. Slight distortion is observed in the system that becomes more noticeable during the third H_2_ encapsulation which slowly decreases for the fourth and the fifth hydrogen molecule encapsulation. This is reflected in the slightly conflicting trend in the corresponding interaction energies. Now in the case of 5^12^6^4^ clathrate, obtaining the minimum energy structure was difficult. It is fascinating to note that the encapsulation of one H_2_ into the cage stabilizes the structure although it could not provide with the minimum energy structure. Further incorporation of guest molecules deforms the structure of the system. For 5^12^6^8^, the interaction energy for all the six H_2_ encapsulation is negative making the process favorable. The large size of the host cavity makes it feasible to accommodate all the six guest molecules efficiently. Positive ∆G value suggests that the complexes are kinetically stable. Finally, it can be concluded that the 5^12^ and 5^12^6^2^ clathrates can encapsulate up to two hydrogen molecules without undergoing any structural distortions, whereas the 5^12^6^8^ clathrate may entrap up to six H_2_ molecules depending upon the level of theory used. Calculation of CDFT-based reactivity descriptors of the complexes with and without H_2_ encapsulation suggests that for most of the systems, stability increases with the increase in number of trapped hydrogen molecules. This is concluded from the increasing hardness and decreasing electrophilicity values.

**FIGURE 13:**
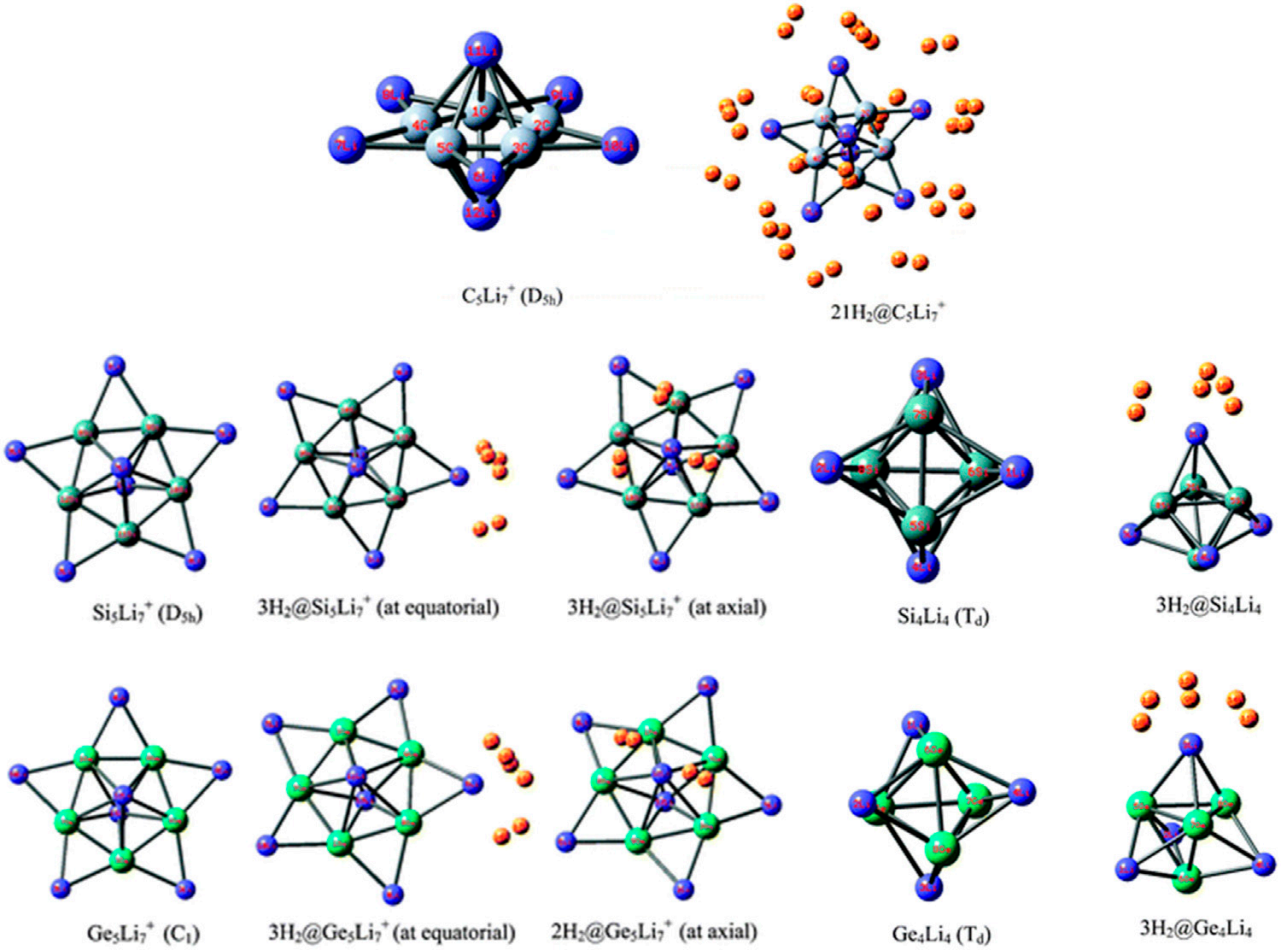
Optimized structures of C_5_Li_7_^+^, M_5_Li_7_^+^, M_4_Li_4_ (M = Si, Ge) and their H_2_-trapped analogues at the M06/6-311+G(d,p) level. (Adapted from Pan et al., 2012b with permission from the PCCP Owner Societies.).

**FIGURE 14:**
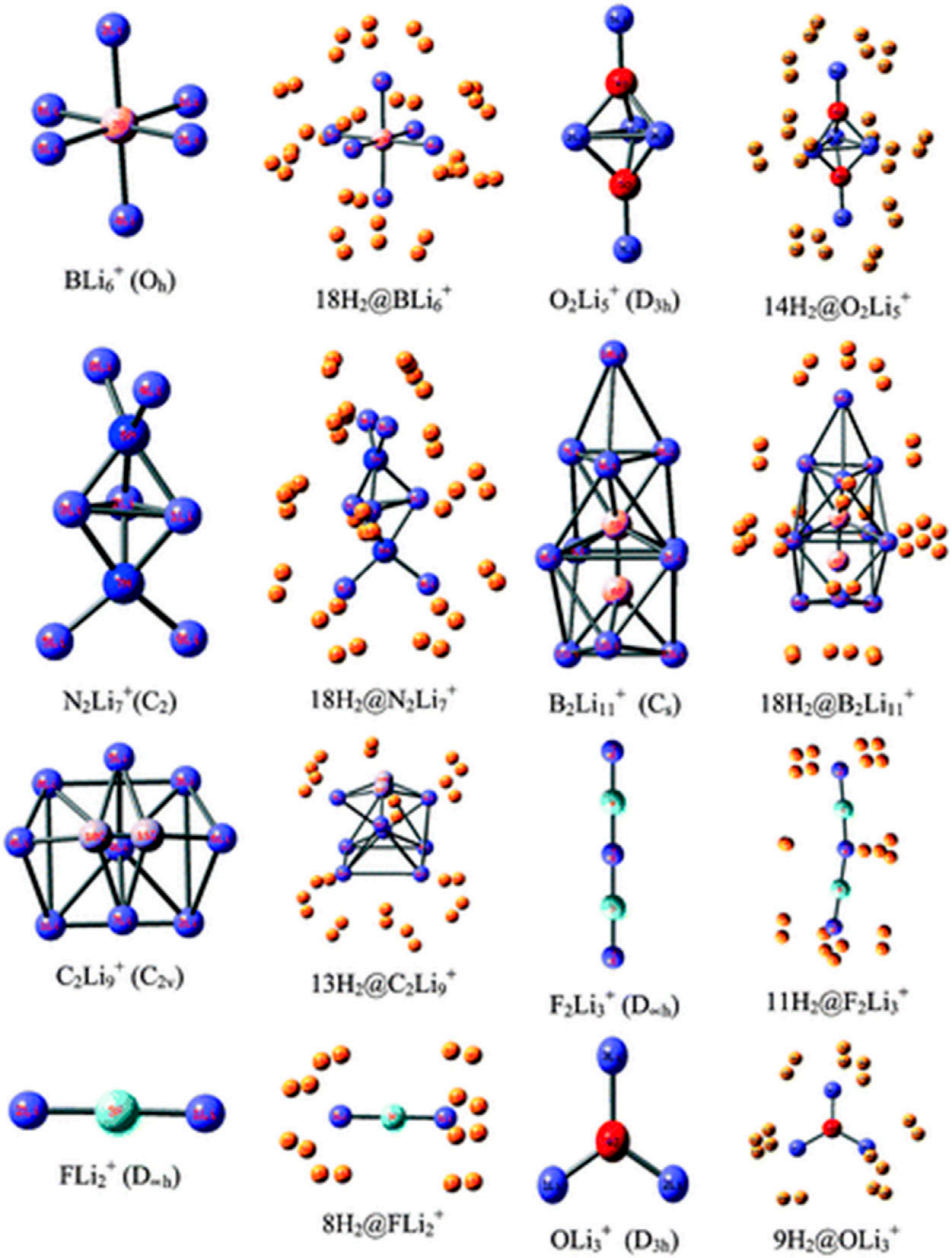
Optimized structures of studied super-alkali ions and their hydrogen-trapped analogues at the M052X/6-311+G(d) level. (Adapted from Pan et al., 2012b with permission from the PCCP Owner Societies.).

Li ion is popularly known to bind well with hydrogen molecule owing to its positive charge (Pan et al., 2012a). Inspired by this, a number of efforts have been made to effectively polarize the Li center of various clusters to increase its hydrogen adsorbing ability. Here we study the H_2_ storage potential of the Li-doped clusters, M_5_Li_7_^+^ (M = C, Si, Ge), M_4_Li_4_ (M = Si, Ge) at the M06/6-311+G(d,p) level, and some super-alkali ions at the M052X/6-311+G(d) level (Figures 13, 14). The Li centers attain a net positive charge due to the high polarizability of the clusters, facilitating electrostatic interactions to bind with the H_2_ molecules. The negative values of interaction energies and enthalpies indicate the efficacy of these clusters to be good H_2_ storage materials. The gravimetric wt% of adsorbed H_2_ are 28.0, 18.3, 9.3, 14.7, and 7.1 for C_2_Li_7_^+^, Si_5_Li_7_^+^, Ge_5_Li_7_^+^, Si_4_Li_4_, and Ge_4_Li_4_, respectively. For the super-alkali ions, the values range from 13.2 to 40.9%, with the highest being that for BLi_6_^+^. On applying electric field, a gradual improvement is observed in the interaction energy value. Thus, in terms of gravimetric wt%, BLi_6_^+^ is preferable whereas the interaction energy per H_2_ molecule suggests B_2_Li_11_^+^ to be the preferred choice for hydrogen storage.

### (HF)_2_ Confinement in Fullerene Cages

**FIGURE 15:**
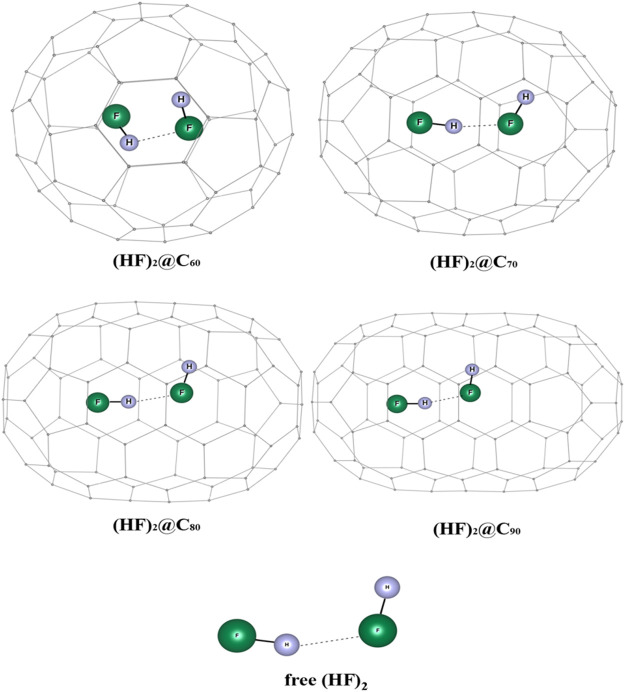
Optimized structures of (HF)_2_@C_*n*_ (*n* = 60, 70, 80, 90) and free (HF)_2_ at ωB97X-D/6-31G level. (Reprinted from Khatua et al., 2014b with permission from Elsevier. Copyright© 2014, Elsevier B.V.).

The influence of encapsulation on the hydrogen bond strength in (HF)_2_ within the fullerene cages is studied using DFT and *ab initio* MD (Khatua et al., 2014b). The optimized geometries of (HF)_2_@C_*n*_ (*n* = 60, 70, 80, 90) complexes wB97X-D/6-31G are depicted in Figure 15. The dissociation energy, enthalpy, and change in free energy are negative for the (HF)_2_@C_60_ system which indicates that the encapsulation process is thermodynamically unfavorable, whereas positive values for the rest of the HF encapsulated C_n_ cages imply them to be favorable (highest being for the C_80_ cage). Owing to the smaller size of the C_60_, the HF units orient themselves antiparallelly to reduce repulsion at the cost of hydrogen bond strength. Thus, the energy associated with the HF-HF interaction is observed to be highest in the C_60_ cage (positive, and hence repulsive in nature). For all the studied cases, upon encapsulation, the hydrogen bond distance reduces from that in the free state, the least being inside the C_70_ cage. The EDA study reveals that the contribution from ∆*E*_pauli_ increases and the ∆*E*_int_ value decreases with decreasing the C_*n*_ cage cavity except for C_80_ cage. For the C_60_ cage, a very large value of ∆*E*_pauli_ makes the overall ∆*E*_int_ value positive. On account of the smaller H-bond distance within the C_70_ and C_90_ cages compared to the same within the C_80_ cavity, both the ∆*E*_elstat_ and ∆*E*_orb_ contribute more to the attractive interaction than those in C_80_. AIM analysis reveals that for all these confined systems, ∇^2^ *ρ*(r_c_) > 0 and H(r_c_) < 0 implying the partial covalent nature of the hydrogen bonds. The hydrogen bond is mostly covalent in case of (HF)_2_@C_70_ ELF analysis that also supports this observation.

## Concluding Remarks

There exists an appreciable amount of interest in the field of cluster chemistry, especially in the gas-phase and surface-adsorbed studies, and for good reasons. Common curiosities in this area include the difference between the properties exhibited by the bulk and individual clusters, how the cluster size affects the overall behavior of the bulk, *etc*. Other important branches of this cluster chemistry include solving their global optimization problem in a fast and cost-effective way, and investigating the effect of confinement on the cluster-encapsulated systems.

The global optimizers discussed in this review are shown to locate the global minimum configurations for small metallic and nonmetallic clusters with less execution time and higher success rate than commonly used optimization algorithms, without having the need to impose any symmetry constraint or any other external restrictions. The only requirement is to adjust the local and global best parameters at each iteration. Comparisons made between our modified PSO with other DFT-integrated BH and SA reveal the superiority of the former with respect to the total execution time and number of iterations the program takes to converge. Again, the DFT-integrated FA turns out to be more efficient than the modified PSO. Furthermore, the ADMP-CNN-PSO technique is well suited for locating the global solution from a huge dataset of initial configurations.

The effect of adsorption and confinement of hydrogen, noble gas atoms, and various other small molecules on their stability, reactivity, nature of interactions, and dynamics are studied from a DFT perspective. The concept of aromaticity is analyzed in terms of CDFT-based descriptors such as *E*, *α*, *ω*, and *η*, where a lower value of the first three parameters and a higher value of hardness in comparison with that of a reference system characterize an aromatic molecule. The reverse is true for antiaromatic compounds. Certain guest@host complexes containing loosely bound electrons acting as anions and showing high NLO properties, known as molecular electrides, are capable of bond activation in small molecules. Other host–guest complexes exhibit fluxionality. One such example is the B_40_ cage whose fluxional property remains unaltered even after Ng atoms encapsulation. The complexation ability of the B_40_ cage is also studied in some sandwich complexes and it is seen than the presence of Xe within the cage enhances its complexation ability. The gas molecules accommodated within the Octa acid cavitand become slightly more reactive compared to their free state. Most of the OA-guest complexes are stable with respect to dissociation. OA can thus be designated as a reasonably good storage material for a variety of small gas molecules. Cucurbiturils form another class of compounds which is well known for its hosting capabilities. CB[6] can act as an efficient noble gas carrier and CB[7] can bind up to 52 hydrogen molecules (8.3 wt%). CB[7] is also found to be highly selective toward the adsorption of SO_2_ and hence can be used in separating SO_2_ from a gas mixture. It is also known to accelerate the otherwise slow [4+2] cycloaddition reaction. The binding ability of the transition metal boron cluster (MB_12_^-^) with isoelectronic species, CO and N_2_, is studied along with its fluxionality. Bond activation in both CO and N_2_ is observed, and the rotation of the ligand-bound complex makes it look like a spinning umbrella. Hydrogen storage capabilities of clathrate hydrates, Li-doped clusters, and super alkali are investigated and it is found that the former can accommodate 2–6 hydrogen molecules, whereas the Li systems show a gravimetric wt% range of 7.1–28.0% for the star-like clusters and 13.2–40.9% for the super-alkali systems. The (HF)_2_ encapsulation by the fullerene cages describes the confinement effect on the H-bond therein. Apart from C_60_, all the cages form the complexes in a thermodynamically favorable process. Also, a partial covalent character is observed in the H-bonds upon confinement.
